# Comprehensive analysis on the regulatory mechanism of active ingredient accumulation during fermentation process of Massa Medicata Fermentata: microbe and metabolic profiles

**DOI:** 10.3389/fmicb.2025.1548427

**Published:** 2025-03-12

**Authors:** Yun Li, Ya-Juan Wang, Xiao-Peng Guo, Hong-Yuan Zhao, Hai-Wei Ren, Hong-Yu Li

**Affiliations:** ^1^School of Pharmacy, Lanzhou University, Lanzhou, China; ^2^Lanzhou Institute for Food and Drug Control, Lanzhou, China; ^3^School of Energy and Power Engineering, Lanzhou University of Technology, Lanzhou, China; ^4^School of Life Science and Engineering, Lanzhou University of Technology, Lanzhou, China

**Keywords:** Massa Medicata Fermentata, fermentation process, microbial community succession, widely targeted metabolomics, regulatory mechanisms

## Abstract

**Background:**

Massa Medicata Fermentata (MMF) is a traditional medicinal/edible fermented product; however, comprehensive research on the fermentation process from a microscopic perspective remains limited. In this study, we aimed to investigate the dynamic changes and correlations of physicochemical properties, microbial communities, and metabolite profiles in different fermentation stages (0, 48, 72, and 96 h) of MMF.

**Methods:**

Standard analytical tests, microbiome sequencing, broad-target metabolism, mixed standard-based mass spectrometry, and fine structure analysis were integrated to elucidate fluctuations in physicochemical, microbial, and metabolic levels during MMF fermentation.

**Results:**

During the fermentation process, bacterial diversity generally shows an increasing trend, whereas fungal diversity generally shows a decreasing trend. Revealing that the differentially abundant metabolites were primarily categorized into lipids, amino acids and derivatives, phenolic acids, organic acids, flavonoids, lignans and coumarins, nucleotides and derivatives, and alkaloids. Structural equation modeling and correlation analysis indicated that two species of bacteria (*Bacillus velezensis*, *Bacillus safensis*) and four species of fungi (*Apiotrichum montevideense*, *Geotrichum bryndzae*, f_Dipodascaceae, *Saccharomycopsis fibuligera*) showed significant positive correlations with five types of differential metabolites, including lipids, flavonoids, phenolic acids, lignans and coumarins, and organic acids. These differential metabolites are essential components responsible for the therapeutic effects of MMF, particularly those that reach peak concentrations at 72 h of fermentation.

**Conclusion:**

These findings are expected to provide a reference for developing strategies to strengthen the quality of MMF and promote its modern application.

## 1 Introduction

The production of traditional foods and Chinese medicines is intertwined with the history of fermentation technology. Numerous foods and Chinese medicines are produced by fermentation (Li et al., 2020). Advances in fermentation technology have made fermentation an indispensable part of the processing of traditional Chinese medicine, and numerous varieties of fermented medications have been developed (Zhang et al., 2022). The 2020 edition of the People’s Republic of China Pharmacopoeia indicates that proprietary Chinese medicine prescription comprises fermented traditional Chinese medicines, which hold a significant market share among these. Massa Medicata Fermentata (MMF), first documented in the Tang Dynasty’s “Treatise on Medicinal Properties,” has been extensively used in traditional Chinese medicine because of its distinctive composition and pharmacological activity (Xu et al., 2019). MMF is crafted by blending an assortment of natural food and medicinal ingredients in precise proportions, followed by natural fermentation, dicing, and drying (Bai et al., 2023; Liu et al., 2023a,b). During fermentation, microbial communities experience succession, accompanied by changes in their metabolic functions. For instance, microorganisms secrete digestive enzymes such as cellulases, lipases, and proteases, which facilitate the breakdown of complex chemical components present in feedstock, resulting in the production of various bioactive metabolites, including fatty acids, amino acids, saccharides, acylglycerols, and flavonoids (Liu et al., 2017). Because of the abundance of active components, MMF plays a pivotal role in regulating immunity, antioxidation, anti-inflammation, energy metabolism, and signal transduction, rendering it a valuable therapeutic option to treat metabolic diseases, such as gastrointestinal diseases, hyperlipidemia, and obesity (Roh et al., 2015; He et al., 2020). MMF is referenced in numerous renowned traditional Chinese medicine texts, including “Qi Min Yao Shu” (Jia, S. X., 533–544 CE), “Treatise on Chinese Herbal Nature” (Zhen, Q., Tang Dynasty, 618–907 CE), and “Seeking Truth from Materia Medica” (Huang, G. X., Qing Dynasty, 1769) (Fan et al., 2024). Besides its use as a medicinal material, MMF can be consumed as folk food over an extended period, such as in tea or porridge, serving the dual purpose of a medicinal food (Liu et al., 2021; Bai et al., 2023). For example, MMF tea can effectively improve spleen and stomach weakness and food accumulation, showing obvious beneficial effects (Liu et al., 2023a,b; Chen et al., 2023).

With the accumulation of experience and advances in science and technology, research on the pharmacological efficacy of MMF and its clinical applications has become increasingly sophisticated, which has led to sharp growth in the demand for MMF in several countries (Wang et al., 2020; Fu et al., 2023). Nevertheless, prevailing production techniques for MMF predominantly rely on conventional natural fermentation methods. Despite their prevalence, traditional fermentation techniques are subject to significant constraints, with inevitable variations in the quality of the raw materials used during the preparation process (Gao et al., 2023). Additionally, dynamic control of temperature and humidity, reliance on subjective assessments of fermentation endpoints, and quality evaluations based on macroscopic characteristics (e.g., color, colony morphology, surface texture, pore structure, and odor) pose significant limitations to current production methods. Consequently, this leads to considerable inconsistencies in product appearance and intrinsic quality, which hampers clinical application (Chen et al., 2020; Shi et al., 2023). It is imperative to comprehensively research microorganisms, primary and secondary metabolites, catalytic enzymes, and inorganic elements from the micro perspective of the fermentation process. This will facilitate the establishment of accurate quality standards and enhance the stability, efficacy, and safety of MMF in clinical applications. In recent years, numerous scholars from domestic and international academic institutions have investigated this phenomenon. Research indicates that changes in digestive enzyme activity during MMF fermentation can serve as a key indicator for regulating fermentation and assessing quality (Xu et al., 2012). Throughout the fermentation process, the concentrations of certain substrate components, such as ferulic acid and caffeic acid, gradually decreases, while metabolites like lactic acid, which are initially absent, significantly increase (Zhang et al., 2022). Analysis of volatile substances reveals that the levels of primary components declines in the early stages of fermentation, whereas other flavor compounds, including benzyl alcohol, acetic acid, and methyl phenylacetate, rise notably, indicating significant changes in flavor composition (Yin et al., 2024). Furthermore, analysis of microbial community structure demonstrates that dominant species shift and diversity decreases in the later stages of fermentation, underscoring the close relationship between microbial succession and the fermentation process (Chen et al., 2020).

The current research primarily focuses on a single-dimensional exploration of the MMF fermentation process at the micro level, which includes changes in physiochemical properties (Zhang et al., 2022; Liu et al., 2023a,b), the succession of microbial communities (Xu et al., 2013), and the generation of active ingredients (Xu et al., 2019). Although multiple indicators exhibit significant changes, studies conducted within a single dimension often lack sufficient information to detail the impact of independent variables and their interactions on fermentation quality. This limitation not only hinders our understanding of the quantitative evaluation of the effects of key indicators and the mechanisms by which they influence the fermentation process, but also impedes the precise regulation of microecology based on bioaugmentation and biostimulation. MMF fermentation is a microbe-driven material conversion process in which active products generate feedback and regulate microbial activity, forming a complex mechanism involving interactions among physicochemical properties, microorganisms, and active components. The composition of MMF changes over time during fermentation, resulting in variations in pharmacological quality. During the transition from raw materials to MMF, microorganisms play a crucial role in fermentation control. Creating a synthetic microbial community can greatly enhance product quality and production stability and reduce the need for specific environmental conditions in solid-state fermentation of edible and medicinal koji. However, its application in MMF requires the accumulation of more foundational data. First and foremost, the acquisition and application of functional species must be grounded in a deep understanding of the complete microecological structure and function of MMF, centered on microorganisms. Therefore, it is imperative to study the composition of MMF and its key active components at different times to control and evaluate the quality of the fermentation process. The integration and application of multi-omics technologies provide researchers with powerful tools for this research area (Liu et al., 2021). Nevertheless, few studies have concurrently investigated the MMF fermentation process from diverse viewpoints, including physicochemical properties, microorganisms, and active components, and elucidated their interrelationships.

In this study, we first optimized the key environmental parameters of the traditional fermentation process and established a precise temperature and humidity control system to ensure stable production of MMF. Building on this foundation, we will systematically analyze the physicochemical properties, microbial community structure, and dynamic evolution of metabolites at different fermentation stages to elucidate the dynamic characteristics and quality formation mechanisms of the fermentation process. Additionally, we plan to validate our strategy through multidimensional data to ensure the scientific reliability of metabolite data and construct an association network model to preliminarily clarify the relationship between key microorganisms and differential metabolites during MMF fermentation. This study aims to decode the internal material transformation modes of traditional fermentation processes, promote the establishment of a theoretical framework for quality formation mechanisms, and ultimately provide a scientific basis for optimizing fermentation processes and improving the quality standards of traditional Chinese medicine fermentation preparations. Collectively, these efforts are expected to offer valuable insights for the modernization and advancement of traditional fermentation technologies.

## 2 Materials and methods

### 2.1 Fermentation of MMF and sample preparation

The traditional fermentation process of MMF was conducted in accordance with the “Ministry of Health Drug Standards Traditional Chinese Medicine Formulation” (Chen et al., 2020). We extensively monitored environmental temperature and humidity during the optimal fermentation period. The Box–Behnken design response surface methodology was employed to optimize the fermentation conditions, determining the optimal temperature and relative humidity for MMF fermentation to be 32°C and 77.0% RH, respectively. Consequently, we replaced open natural fermentation with controlled fermentation in a constant temperature and humidity chamber to ensure the reliability and reproducibility of the experimental results (Supplementary Figure S1). The specific preparation process is: we prepared MMF by grinding *Semen Armeniacae Amarum* and *Semen Vignae* into coarse powder, weighing 100 g of each, and mixing them with 2,500 g of flour and 5,000 g of wheat bran in a trough-type mixer. Subsequently, 500 g of *Herba Artemisiae Annuae*, *Herba Polygonum Hydropiper*, and *Herba Xanthii* were collected, an appropriate amount of water was added, and the mixture was boiled for 1 h, before filtering, concentrating, and mixing with powdered medicinal herbs, while stirring well. The obtained MMF sample was placed in a constant temperature and humidity chamber for fermentation. Samples were taken at the following timepoints to monitor the progress of the fermentation process: 0 h (T0 h), 24 h (T24 h), 39 h (T39 h), 48 h (T48 h), 63 h (T63 h), 72 h (T72 h), and 96 h (T96 h). Each sample was mixed and stored at −80°C. *Semen Armeniacae Amarum*, *Semen Vignae*, and *Herba Artemisiae Annuae* were purchased from Beijing Tongrentang Lanzhou Pharmacy Co., Ltd.; flour and wheat bran were purchased from Shanxi Province, China; *Herba Polygonum Hydropiper* was collected from Qinzhou City, Guangxi Province, China; and *Herba Xanthii* was collected from Lanzhou City, Gansu Province, China.

### 2.2 Determination of physicochemical properties

#### 2.2.1 Detections of pH, moisture, and inorganic element content

The pH values of MMF samples at different fermentation times were determined according to “The People’s Republic of China Pharmacopoeia 2020” four general 0631 pH measurement method, Part IV (Wu et al., 2022). The moisture content was measured according to “The People’s Republic of China Pharmacopoeia 2020” general principles 0832, second method: drying method (Zhao et al., 2024); and the content of inorganic elements was determined using inductively coupled plasma mass spectrometry (Li et al., 2024).

#### 2.2.2 Measurements of free amino acid content

To prepare individual stock solutions at a concentration of 1 mg/mL for each amino acid, 19 amino acids were used. A mixed standard working solution with a concentration range of 1.0–5.0 μg/mL was created from these stock solutions. After appropriate dilution, the samples were analyzed using UPLC-MS/MS to obtain a calibration curve. Briefly, 0.25 g was weighed and added to 20 mL of a 0.2% formic acid aqueous solution (v/v). Ultrasonication (power, 300 W; frequency, 40 kHz) was performed for 30 min. Subsequently, the solution was centrifuged at 10,000 rpm for 10 min, and 1 mL of the supernatant was added to a 10 mL volumetric flask, mixed with 0.2% formic acid aqueous solution (V:V) up to the mark, vortexed for 1 min, and then filtered through a 0.22 μm membrane. The chromatographic conditions were as follows: chromatographic column: InfinityLab Poroshell 120 HILIC-Z (2.1 × 100 mm, 2.7 μm); mobile phases: A, 10% (200 mM ammonium formate aqueous solution, pH = 3)/90% water; B, 10% (200 mM ammonium formate aqueous solution, pH = 3)/90% acetonitrile; gradient elution: 0–10 min, 100–70% B; 10–11 min; flow rate, 0.8 mL/min; column temperature, 35°C; and injection volume, 0.3 μL. The mass spectrometry conditions were as follows: electrospray ionization source in positive ion mode (ESI^+^); d-MRM scanning mode, nebulizing gas, N_2_; drying gas flow rate, 11.0 L/min; spray voltage, 500 V; gas flow temperature, 325°C; capillary voltage, 4,000 V; sheath gas flow rate, 12 L/min; and sheath gas temperature, 350°C.

#### 2.2.3 Assessment of total phenolic acids, flavonoids, and coumarin content

The total phenolic acid, flavonoid, and coumarin contents of the MMF samples were determined at different fermentation stages using spectrophotometry. Standard solutions of 1,3-O-dicaffeoylquinic acid, chrysosplenol D, and scopoline were prepared by dissolving them in methanol to concentrations of 0.99176, 661.5, and 1.2892 mg/mL, respectively. The absorbances of these standard solutions were measured at 317, 354, and 344 nm to plot the standard curves. The MMF sample (0.5 g) was weighed and treated with 25 mL of methanol, 20 mL of 50% ethanol, and 40 mL of 50% ethanol, before subjecting to ultrasonication for 30 min and centrifugation at 9,000 rpm for 8 min. Finally, the supernatant was collected as the test solution, and absorbance values were measured.

### 2.3 Microbial diversity analysis

DNA was extracted from each sample using a Fast DNA Spin Kit. The DNA integrity was assessed using 1% agarose gel electrophoresis, and the purity and concentration of total DNA were measured using a Nanodrop 2000 spectrophotometer. After DNA testing, bacterial 16S rDNA V3–V4 regions were amplified using the universal primers 338F and 806R, and fungal internal transcribed spacers (ITS) were amplified using the primers ITS1F and ITS2R. The PCR products were purified, quantified, and homogenized, and the quality and quantity of the final products were assessed. Paired-end sequencing was performed on an Illumina MiSeq PE300 platform. Raw sequences were quality-controlled, merged, and filtered using Fastp (version 0.19.6) (Chen et al., 2018), Flash (version 1.2.11) (Magoč and Salzberg, 2011), and Uparse (version 11) (Caporaso et al., 2011) to obtain valid sequences. Based on 97% similarity, operational taxonomic unit (OTU) clustering was performed on the sequences using the USEARCH7-uparse algorithm, and chimeras were removed. The taxonomic classification of OTUs for bacteria was conducted using the Ribosomal Database Project (RDP, version 11.5) in conjunction with the Silva 138 16S species classification database. The fungal taxa were classified using the UNITE database (version 8.0). Taxonomic annotations were made with a confidence threshold of 70%, and the community composition of each sample was analyzed at various taxonomic levels.

### 2.4 Broad-target metabolomics detection and analysis

MMF samples with different fermentation times were placed in a freeze dryer (Scientz-100F) for vacuum freeze-drying, before grinding into powder using a grinder (MM 400, Retsch). A 100 mg aliquot of the powder was dissolved in 1.2 mL of 70% methanol extraction solution and incubated overnight at 4°C. The mixture was then centrifuged at 12,000 rpm for 10 min, and the supernatant was filtered through a micropore filter membrane (0.22 μm pore size) to obtain the sample. UPLC and MS/MS were used for data acquisition. The liquid chromatography conditions were as follows: Column, Agilent SB-C18 (1.8 μm, 2.1 mm × 100 mm); mobile phase A, ultra-pure water with 0.1% formic acid, and phase B, acetonitrile with 0.1% formic acid; flow rate, 0.35 mL/min; column temperature, 40°C; and injection volume, 4 μL. The mass spectrometry conditions were as follows: ESI source parameters included an ion source with turbo spray; source temperature at 550°C; ion spray voltage set at 5,500 V for positive mode and −4,500 V for negative mode; ion source gases I, II, and curtain gas set to 50, 60, and 25.0 psi, respectively; and collision-induced dissociation parameters set to high. Instrument tuning and mass calibration were performed using 10 and 100 μmol/L polypropylene glycol solutions in triple quadrupole (QQQ) and LIT modes. QQQ scanning was performed in multiple reaction monitoring (MRM) mode with a collision gas (nitrogen) set in the medium. Qualitative scoring methods were used to filter out metabolites with large mass deviations from parent ions. The mass deviation of the parent ions for the key metabolites did not exceed 15 ppm. The DP and CE of each MRM ion pair were determined by further optimization. For each period, a specific set of MRM ion pairs was monitored based on the eluted metabolites. Identification was carried out using a self-built database, the Metware Database, by aligning secondary spectral information, such as collision energy, retention time, and m/z ratio, with the target ion pair information. Normalization was performed by dividing the peak area of each metabolite by the total peak area of the sample and then multiplying by the average peak area of all samples.

### 2.5 Verification of 12 compounds by UPLC-MS/MS

Appropriate amounts of each analyte reference standard were precisely weighed and dissolved separately in methanol to prepare a single reference standard stock solution. From these reference standard stock solutions, mixed reference standard solutions were prepared by diluting with methanol to achieve final concentrations of 41.98 μg/mL for amygdalin, 10.25 μg/mL for caffeic acid, 75.25 μg/mL for scopoletin, 18.90 μg/mL for vitexin, 9.19 μg/mL for hyperoside, 10.10 μg/mL for isochlorogenic acid B, 9.73 μg/mL for isochlorogenic acid A, 9.57 μg/mL for isochlorogenic acid C, 10.01 μg/mL for quercetin, 9.49 μg/mL for apigenin, 19.71 μg/mL for eupatilin, and 8.74 μg/mL for chrysosplenetin. Next, 1.0 g of powder from different fermentation stages of the MMF sample was weighed and placed in a conical flask containing 20 mL of methanol. The mixture was then ultrasonicated (300 W, 40 kHz) for 10 min and then centrifuged at 10,000 rpm for 5 min. The supernatant was collected and filtered through a 0.22 μm membrane filter to obtain the test solution. Next, both the mixed reference standard solution and MMF test solution were aspirated and analyzed under chromatography-mass spectrometry conditions. The mass deviations of the parent ions of the 12 compounds were not set to exceed 15 ppm. The chromatographic conditions were as follows: Column, Brownlee SPP C18 (2.1 mm × 100 mm, 2.7 μm); mobile phase, 0.1% formic acid and 5 mmol L^−1^ ammonium acetate aqueous solution (A)—acetonitrile (B); gradient elution, in negative ion mode, 0–3 min, 90–85% A; 3–5 min, 85% A; 5–8 min, 85–30% A; 8–12 min, 30% A; 12–14 min, 30–90% A; 14–16 min, 90% A; flow rate, 0.3 mL min^−1^; column temperature, 35°C; and injection volume, 2 μL. The mass spectrometry conditions were as follows: ESI, scanning in negative ion mode, MRM; dry gas temperature, 340°C; gas flow rate, 11 L min^−1^; nebulizer pressure, 45 psi; and capillary voltage, 3,500 V (ESI^−^).

### 2.6 Correlation analysis

We investigated the correlations and mutual directivity among key microorganisms (12 bacterial and 10 fungal species) that serve as both dominant and indicator species during MMF fermentation. The study focused on physicochemical parameters with VIP values >1 as well as the top 10 upregulated differential metabolites in each category. Pearson correlation coefficients were used to compute the correlation matrix, which was calculated using the “cor” function and visualized with a correlation heatmap created using the “heatmap” package. The heatmap, colored to reveal key correlations, illustrates the relationships between key microorganisms and key physicochemical indicators, as well as important metabolites. Furthermore, principal coordinate analysis (PCoA) and orthogonal partial least squares discriminant analysis (OPLS-DA) were conducted using the R software packages plspm and ggplot2 to visualize the sample grouping. Subsequently, structural equation models were constructed using the “cor.test” function and the “corrplot” package in R to analyze the reciprocal relationships among key microbial species, essential physicochemical indicators, and significant metabolites.

### 2.7 Lipid molecular structure analysis

MMF samples were collected and flash-frozen at various fermentation time points for subsequent analysis. During testing, the samples were removed from the −80°C freezer, thawed on ice, and 200 mg was transferred to 1 mL of water. The mixture was homogenized at 40,000 Hz for 5 min, after which 1 mL of methanol and 2 mL of chloroform were added. The mixture was vortexed for 10 min and then centrifuged at 12,000 rpm for 10 min. The lower layer of the solution was collected, and the extraction was repeated with the upper layer. The two extracts were combined, the solvent was evaporated under nitrogen, and the residue was dissolved in 1 mL of methanol and filtered through a 0.22 μm membrane to obtain the lipid extract. SPLASH^®^ II LIPIDOMIX^®^ mass spectrometry standards (Avanti) were used as isotopic internal standards to prepare a 100 mg/L stock solution of isotopic internal standards. Ten microliters of this stock solution were transferred to 90 μL of lipid extract (with 100 μg/L bovine liver polar lipid extract used as a quality control sample). Structural analysis of lipid types, lipid molecular species, and lipid C=C position isomers was performed using the Structure Precision Reactor Ω Analyzer in conjunction with liquid chromatography and mass spectrometry. In addition, proprietary software and algorithms developed using lipid databases were used to perform statistical analyses based on the relative intensity information of lipid molecular species and the relative intensity information of C=C isomer levels.

### 2.8 Statistical analysis

All experiments were conducted at least three times, and data are reported as the mean ± SD. Statistical analysis of the experimental data was performed using the statistical software SPSS 20. OPLS-DA was used to distinguish intergroup differences.

## 3 Results

### 3.1 Dynamic changes in the macroscopic characteristics of MMF during fermentation

**Figure 1:**
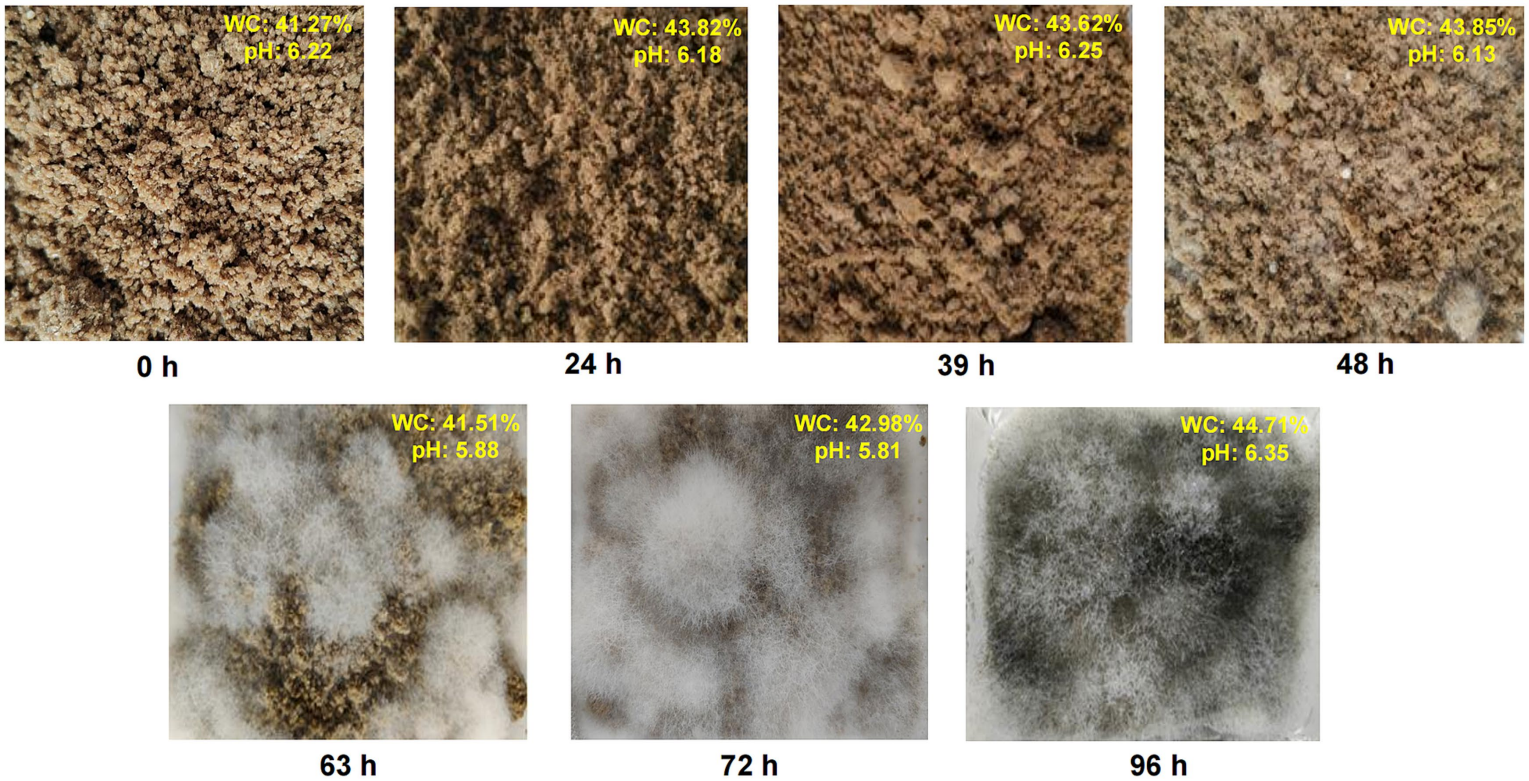
Changes in pH, moisture content, and mycelial growth during the MMF fermentation process.

We conducted a comprehensive study of macroscopic characteristics at different fermentation times, considering mycelial growth, pH, and moisture content during MMF fermentation (Figure 1). Throughout the fermentation process, the softness of the MMF gradually increased, and its color became lighter. Within the first 39 h of fermentation, MMF remained in its original state, with no visible mycelial growth on the surface. At 39 h, white filamentous mycelia began to appear on the surface, and as fermentation continued, the mycelia gradually grew until 72 h when the mycelia basically covered the surface of the MMF, and it was at this time that koji aroma was the strongest on the surface of the material. The color changed from pure white (39 and 48 h) to grayish-white (63 and 72 h). After excessive fermentation for 96 h, the MMF surface became dark gray. The moisture content and pH were measured at different fermentation stages to gain a more comprehensive understanding of the fermentation process. The moisture content initially decreased up to 63 h and then increased over time, reaching a maximum of 44.71% at 96 h. The pH exhibited a decreasing trend up to the optimal fermentation point (72 h), reaching a minimum of 5.81, and then increasing to 6.35 after excessive fermentation.

### 3.2 Dynamic changes in physicochemical properties during MMF fermentation

**Figure 2:**
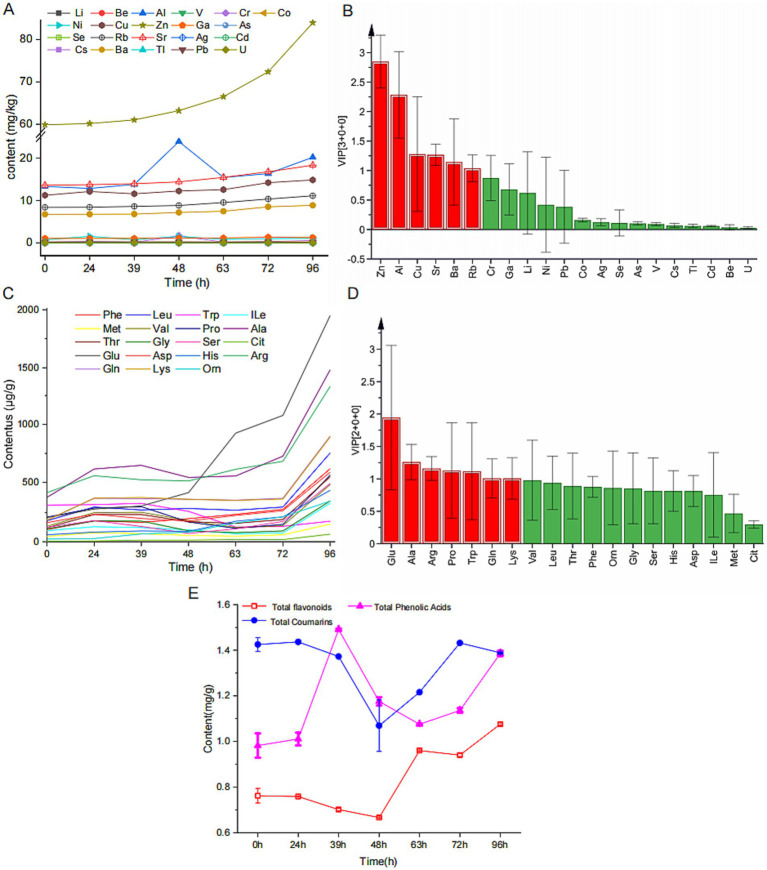
Dynamic changes of the physicochemical properties during MMF fermentation. **(A,C)** Fingerprint profiles of inorganic elements and free amino acids. **(B,D)** VIP values of inorganic elements and free amino acids. **(E)** Changes in total flavonoids, total phenolic acids, and total coumarins.

To clarify the dynamic changes in physicochemical properties during MMF fermentation, we quantitatively analyzed key components such as inorganic elements, free amino acids, total flavonoids, total phenolic acids, and total coumarins. A multi-element fingerprint was established with 21 target inorganic elements on the *x*-axis and the relative content of these elements in each sample on the *y*-axis, as shown in Figure 2A. Zn, Sr, Cu, Al, Rb, and Ba showed increasing trends during fermentation, whereas the other 15 inorganic elements did not exhibit significant changes during the process. A model was established using SIMCA software to study the compositional differences in MMF at different fermentation times (Supplementary Figure S2). This model clearly distinguished MMF from the different fermentation stages, revealing notable compositional differences. Analysis of the VIP plot (Figure 2B) revealed that the main characteristic elements (VIP >1) were Zn, Al, Cu, Sr, Ba, and Rb. Analysis of the dynamic changes in 19 free amino acids at different fermentation stages revealed the content of each amino acid (excluding Glu) followed a trend of first increasing, then decreasing, and finally increasing, the Glu content demonstrated a consistent upward trend throughout the fermentation process (Gao et al., 2023) (Figure 2C). To ensure the stability, validity, and feasibility of the model, a VIP plot (Figure 2D) was constructed, which indicated that the main free amino acids (VIP >1) were Glu, Ala, Arg, Pro, Trp, Gln, and Lys. Analysis of the changes in total flavonoids, phenolic acids, and coumarins at different fermentation stages revealed that both the total flavonoids and coumarins first decreased and then increased, reaching their minimum values at 48 h (0.6664 and 1.1824 mg/g, respectively). In contrast, total phenolic acids initially increased, followed by a decrease, and subsequently increased again during overfermentation, peaking at 39 h with a value of 1.4909 mg/g (Figure 2E).

### 3.3 Changes in microbial community diversity during MMF fermentation

**Figure 3:**
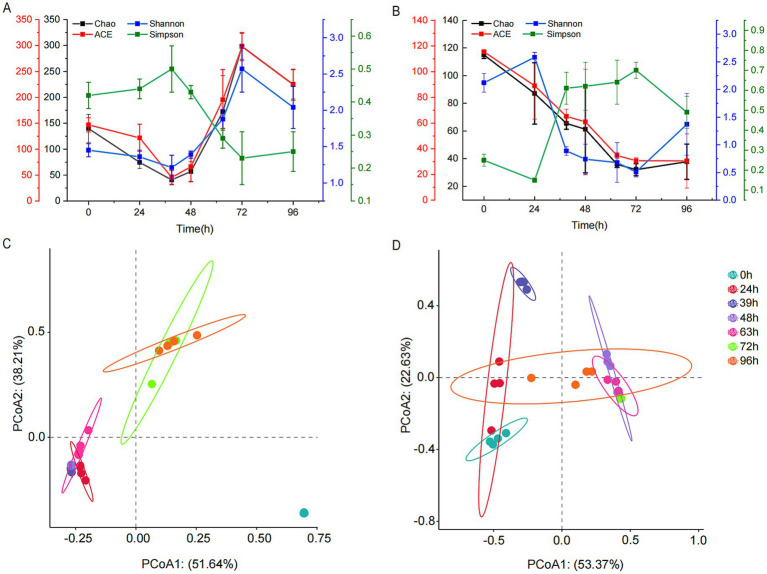
Changes in the microbial community diversity during MMF fermentation. **(A,C)** Bacterial α- and β-diversity. **(B,D)** Fungal α- and β-diversity.

Illumina MiSeq sequencing technology was used to study the dynamic changes in bacterial and fungal communities during MMF fermentation. The number of effective bacterial sequences and the number of effective bases were 1,322,484 and 562,475,493, respectively, with an average length of 425 bp. For fungi, the number of effective sequences and the number of effective bases were 1,470,072 and 386,626,954, respectively, with an average length of 263 bp. The coverage rate for all samples exceeded 0.999, and the dilution curves reached saturation, indicating that the sequencing quality and coverage were sufficient to reflect the complete microbial information of MMF fermentation. The ACE and Chao1 indices represent community richness, whereas the Simpson and Shannon indices represent community diversity. The Simpson index describes species dominance within a community, whereas the Shannon index considers both species richness and evenness. The ACE, Chao1, Shannon, and Simpson indices were used to assess changes in microbial community α-diversity, whereas PCoA was used to evaluate β-diversity. The richness and diversity of the bacterial communities declined from 0 h onwards and reached a minimum after 39 h of fermentation. As fermentation continued, both richness and diversity began to increase, although they decreased again during overfermentation but remained higher than at the beginning of fermentation (Figure 3A). The richness of fungal communities decreased from 0 to 72 h but increased during overfermentation. The diversity increased from 0 to 24 h, decreased from 24 to 72 h, and increased again during overfermentation, although it remained lower than that at the start of fermentation (Figure 3B). In the bacterial PCoA, PC01 and PC02 accounted for 51.64 and 38.21% of the total variance, respectively (Figure 3C). The plot shows positive cohesion within each group. For the 24, 39, 48, and 63-h groups, the distances between samples were relatively small, showing some overlap and indicating similar species composition. Similarly, the 72-h and 96-h groups also had small distances between samples with some overlap, reflecting similar species compositions. However, all groups were distinctly separated from the 0-h samples, demonstrating good clustering between groups and significant differences in species composition. For the fungi, PC01 and PC02 accounted for 53.37 and 22.63% of the total variance, respectively (Figure 3D). Each group exhibited good cohesion. At 0, 24, and 39 h, the samples at each time point were not fully separated, indicating poor clustering and similar species compositions. At 48, 63, and 72 h, the samples at each time point also showed incomplete separation and poor clustering with similar species compositions. However, there was a high degree of separation between the two major groups, and the 96-h samples were interspersed between the two groups.

### 3.4 Structural compositional succession of MMF fermentation microbial communities

**Figure 4:**
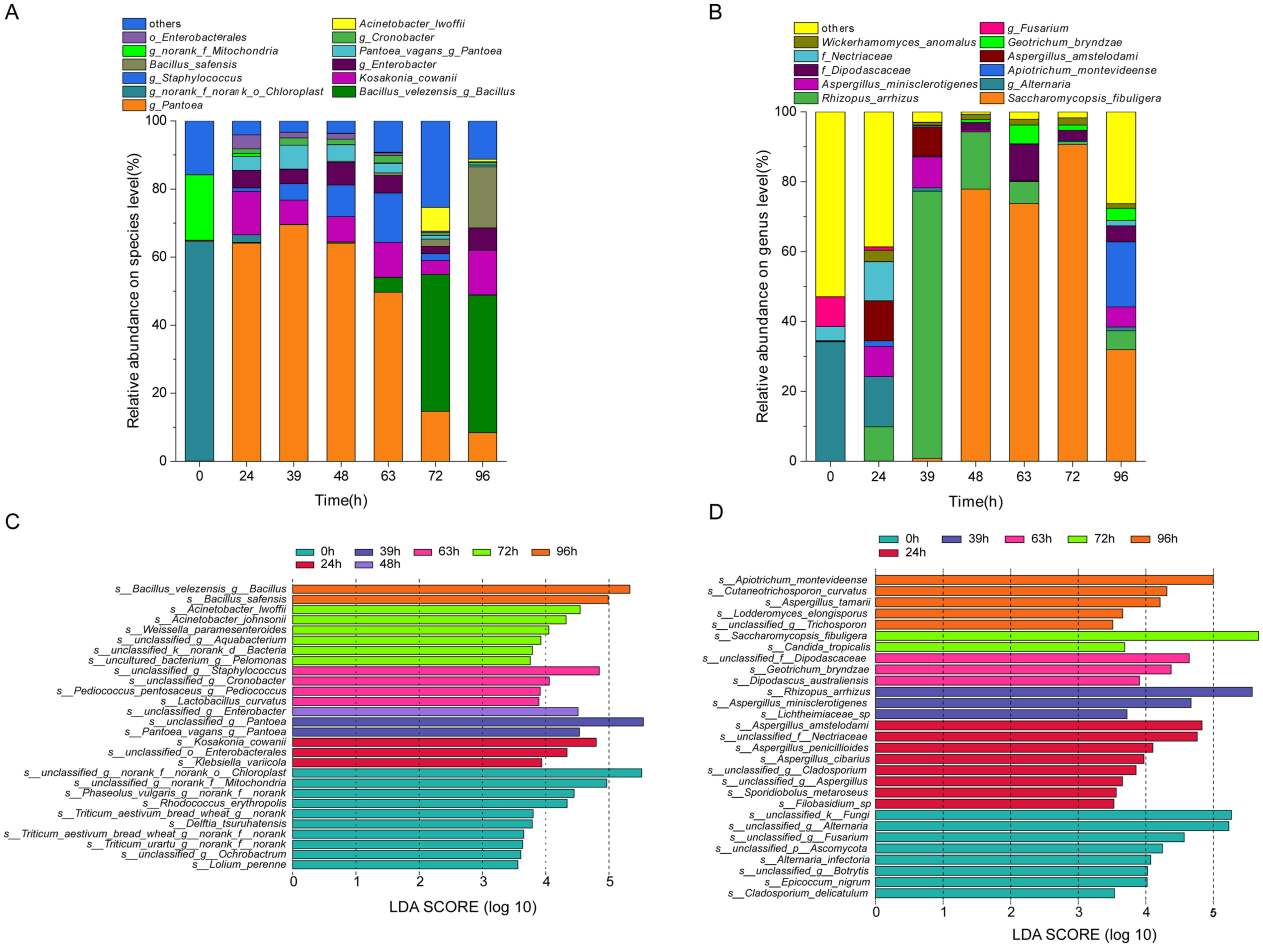
Succession of microbial community composition during MMF fermentation. **(A,C)** Dominant and indicator species for bacteria. **(B,D)** Dominant and indicator species for fungi.

In total, 527 genera were detected in the analysis of the bacterial community structure, including 10 dominant genera with an average relative abundance of >1% (Supplementary Figure S3). In addition, 764 species were identified, of which 12 were dominant (average relative abundance >1%) (Figure 4A). At the species level, o_Chloroplast and f_Mitochondria had the highest abundances at 0 h (64.59 and 19.23%, respectively). As fermentation progressed, their abundances sharply declined, reaching 0.07 and 0.03% at 39 h and then remained stable. The abundances of *Bacillus velezensis* and *Bacillus safensis* increased, reaching 40.43 and 17.97% at 96 h, respectively. The abundances of *Pantoea* sp., *Staphylococcus* sp., *Pantoea vagans*, *Cronobacter* sp., and o_Enterobacterales exhibited a trend of increasing and then decreasing, peaking at 48, 48, 39, 39, and 48 h of fermentation, with maximum values of 64.08, 9.27, 7.02, 2.12, and 1.67%, respectively; *Kosakonia cowanii*, *Enterobacter* sp., and *Acinetobacter lwoffii* showed a trend of increasing, then decreasing, and increasing again during over-fermentation, reaching their maximum abundances at 96, 48, and 72 h, with values of 13.15, 6.67, and 7.00%, respectively.

A total of 147 genera were detected in the fungal community structure analysis, including 11 dominant genera (average relative abundance >1%) (Supplementary Figure S4). In addition, 217 species were identified, among which 11 were dominant (average relative abundance >1%) (Figure 4B). At the species level, *Alternaria* and *Fusarium* sp. had the highest abundances at 0 h (33.88, 34.15, and 8.55%, respectively). During fermentation, the abundances of certain microorganisms sharply declined before stabilizing. For example, at 39 h, the abundances of *Rhizopus arrhizus*, *Aspergillus minisclerotigenes*, *Apiotrichum montevideense*, f_Dipodascaceae, *Aspergillus amstelodami*, f_Nectriaceae, and *Geotrichum bryndzae* initially increased again. Maximum abundances were reached at different time points, with abundances ranging from 5.43 to 76.43%. In contrast, *Saccharomycopsis fibuligera* showed a continuous increase until 72 h, reaching a maximum abundance of 90.69%, which corresponds to changes in fungal diversity indices. *Wickerhamomyces anomalus* was present throughout fermentation, but did not follow a clear pattern.

Using linear discriminant analysis, specific microorganisms with statistically significant differences between different fermentation periods were identified (LDA score >3.5). At the species level, 28 bacterial species (Figure 4C) and 29 fungal species (Figure 4D) exhibited significant differences in enrichment across different fermentation times (*p* < 0.05). The indicator bacterial species after 0, 24, 39, 48, 63, 72, and 96 h of fermentation were 10, 3, 2, 1, 4, 6, and 2, respectively. Among these, o_Chloroplast and f_Mitochondria at 0 h; o_Enterobacterales and *Kosakonia cowanii* at 24 h; *Pantoea* sp. and *Pantoea vagans* at 39 h; *Enterobacter* sp. at 48 h; *Staphylococcus* sp. and *Cronobacter* sp. at 63 h; *Acinetobacter lwoffii* at 72 h; and *Bacillus velezensis* and *Bacillus safensis* at 96 h were both indicators and dominant species. Indicator fungal species at 0, 24, 39, 48, 63, 72, and 96 h of fermentation were 8, 8, 3, 0, 3, 2, and 5, respectively. Among these species, *Fusarium* sp. and *Alternaria* sp. at 0 h, f_Nectriaceae, and *Aspergillus amstelodami* at 24 h, *Aspergillus minisclerotigenes* and *Rhizopus arrhizus* at 39 h, *Geotrichum bryndzae* and f_Dipodascaceae at 63 h, *Saccharomycopsis fibuligera* at 72 h, and *Apiotrichum montevideense* at 96 h were the indicator and dominant species.

### 3.5 Broad-target metabolomics analysis of MMF fermentation

**Figure 5:**
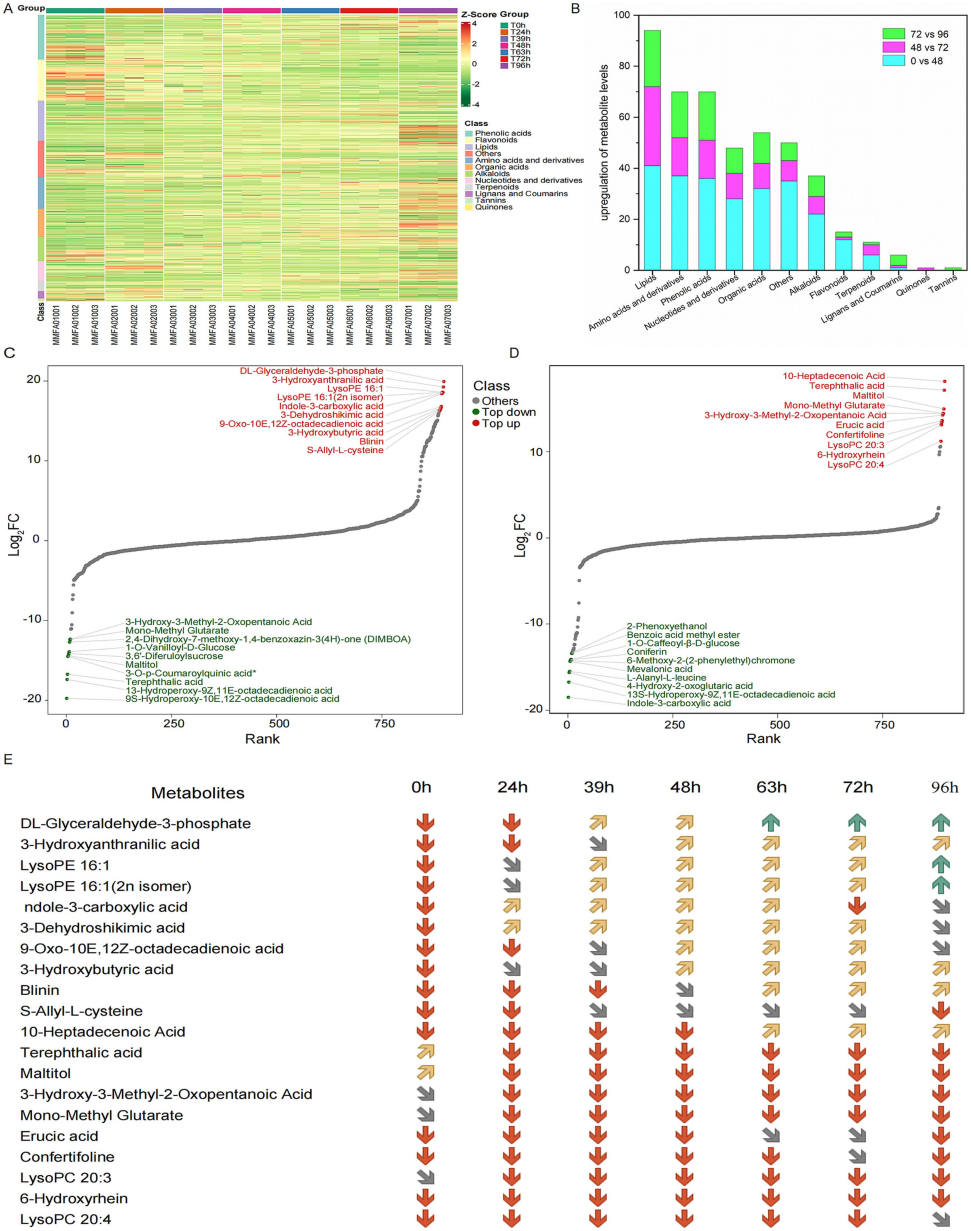
Broad-target metabolomic analysis of MMF fermentation. **(A)** Clustering heatmap of all metabolite contents. **(B)** Classification and trends of differential metabolites for comparisons of 0 vs. 48, 48 vs. 72, and 72 vs. 96 h. **(C,D)** Top ten differential metabolites for the comparisons of 0 vs. 48 and 48 vs. 72 h, respectively. **(E)** The distribution of differential metabolites at each time point. The red, grey, yellow and green arrows represent the relative metabolite contents between 0–10,000, 10,000–100,000, 100,000–1,000,000 and above 1,000,000, respectively.

We next conducted wide-target metabolomic analysis to explore the composition and changes in metabolites during MMF fermentation. Based on HMDB annotations in the positive and negative ion modes, 902 metabolites were identified and classified as phenolic acids, flavonoids, lipids, amino acids and derivatives, organic acids, alkaloids, nucleotides and derivatives, terpenoids, lignans, coumarins, tannins, and quinones. A clustering heatmap of all metabolite contents is presented in Figure 5A. At 0, 24, and 39 h, the metabolites were mainly concentrated in phenolic acids and flavonoids and showed similar distribution patterns. Compared to 39 h before fermentation, the metabolites at 63 and 72 h shifted significantly downward, with a more uniform distribution among lipids, amino acids and derivatives, organic acids, alkaloids, nucleotides, and derivatives. At 48 h, the metabolites were distributed across all categories, representing a transitional state. During overfermentation at 96 h, metabolites were primarily concentrated in lipids, amino acids and derivatives, and organic acids, with the most abundant metabolites constituting a significant proportion. During the fermentation process, the primary metabolites derived from the raw materials decreased significantly, accompanied by a corresponding increase in secondary metabolites. Therefore, 0, 48, 72, and 96 h were selected as the key time points for further studies. To further clarify the changes in metabolites during different fermentation periods, the metabolic components at 0, 48, 72, and 96 h were compared. The differentially expressed metabolites are shown in Figure 5B. The highest number of differential metabolites was observed at 0 vs. 48 h, followed by 72 vs. 96 h, while the lowest number was observed at 48 vs. 72 h. In all groups, lipids accounted for the highest proportion, followed by amino acids and their derivatives, whereas tannins accounted for the lowest proportion. Based on the differential fold changes (log_2_FC values), the top 10 differential metabolites in each group were selected. Differential abundance plots for the top ten metabolites with the highest log_2_FC values are presented for comparison between 0 and 48 h, and 48 and 72 h (Figures 5C,D). The differential metabolites in each group were primarily the lipids and organic acids. Specifically, the log_2_FC values for 0 vs. 48 h ranged from 16.3 to 19.9, with an average of 17.9, whereas for 48 vs. 72 h, the log_2_FC values ranged from 11.22 to 18.16, with an average of 14.5. Among the upregulated differential metabolites, most were potential natural products with biological activities, structural analogs of drug molecules, or important functional molecules in metabolic network nodes (Wang et al., 2024; Jang et al., 2009). Specific changes in the concentrations of the top 10 differential metabolites at each time point are shown in Figure 5E.

### 3.6 Validation of 12 compounds based on UPLC-MS/MS

UPLC-MS/MS is an analytical technique that combines the advantages of UPLC with the high selectivity and sensitivity of MS/MS. It is used to effectively separate and identify structurally similar substances by monitoring the characteristic fragment ions generated by collision-induced dissociation of the mass spectrometry system and has been widely used in the detection and analysis of various metabolites. UPLC-MS/MS was used to measure the concentrations of 12 compounds, including amygdalin, caffeic acid, and scopoletin, at different stages of MMF fermentation. The MRM chromatograms of the mixed standard solutions and samples are shown in Supplementary Figure S5. Targeted qualitative analysis was conducted by precisely matching the retention times and parent ions of each compound in the mixed control solution, and the mass spectrometry parameters of the 12 compounds in non-targeted metabolomics were analyzed. The results showed that in the targeted metabolite analysis, the retention time, parent ion molar mass, fragment ion molar mass, and absolute content of caffeic acid were 2.608 min, 178.90 Da, 134.9 Da, and 10.09 μg/g, respectively. In broad-target metabolomics analysis, the parent ion molar mass, characteristic fragment ions, and relative content were 179.03 Da, 135.05 Da, and 7.77 × 10^6^, respectively. For isochlorogenic acid B, the retention time, parent ion molar mass, fragment ion molar mass, and absolute content in the targeted metabolite analysis were 7.045 min, 515.10 Da, 352.90 Da, and 2.46 μg/g, respectively. In broad-target metabolomics analysis, the parent ion molar mass, characteristic fragment ions, and relative content were 515.12 Da, 353.09 Da, and 7.22 × 10^5^, respectively. Quercetin had a retention time, parent ion molar mass, fragment ion molar mass, and absolute content in the targeted metabolite analysis of 8.117 min, 301.00 Da, 151.00 Da, and 12.03 μg/g, respectively. In broad-target metabolomics analysis, the parent ion molar mass, characteristic fragment ions, and relative content were 303.05 Da, 137.02 Da, and 3.11 × 10^5^, respectively. The mass spectrometry parameters for the remaining nine compounds in both targeted and non-targeted analyses are shown in Table 1. All 12 compounds were detectable by both UPLC-MS/MS and broad-targeted metabolomics analyses, indicating that the results of the broad-targeted metabolomics analysis were accurate and reliable.

**Table 1:** Mass spectrometry parameters of 12 components during MMF fermentation.

Index	Compound	UPLC-MS/MS validation			Broad-target metabolomics	
		Retention time (min)	Parent ion molar mass (Da)	Fragment ion molar mass (Da)	Parent ion molar mass (Da)	Characteristic fragment ions (Da)
mws1580	Amygdalin	2.344	456.10	323.3	456.15	322.8
mws2212	Caffeic acid	2.608	178.90	134.9	179.03	135.05
pme2954	Quercetin	8.117	301.00	151.0	303.05	137.02
Li512115	Isochlorogenic acid B	7.045	515.10	352.9	515.12	353.09
pmp000006	Eupatilin	8.970	343.00	297.9	345.1	330.07
pmn001382	Isochlorogenic acid A	7.255	515.10	352.8	515.12	353.09
pmn001384	Isochlorogenic acid C	7.489	515.10	353.0	515.12	353.09
Lmdp003286	Hyperoside	5.737	463.10	270.9	465.1	303.06
MWS20151	Apigenin	8.399	269.00	117.0	271.06	153.02
MWSHY0096	Chrysosplenetin	9.132	373.10	343.0	375.11	342.07
MWSCX014	Scopoletin	4.855	191.00	176.0	193.05	133.03
MWSHY0181	Vitexin	5.563	431.10	311.1	433.11	313.07

### 3.7 Correlation analysis of physicochemical properties, metabolites, and microorganisms

**Figure 6:**
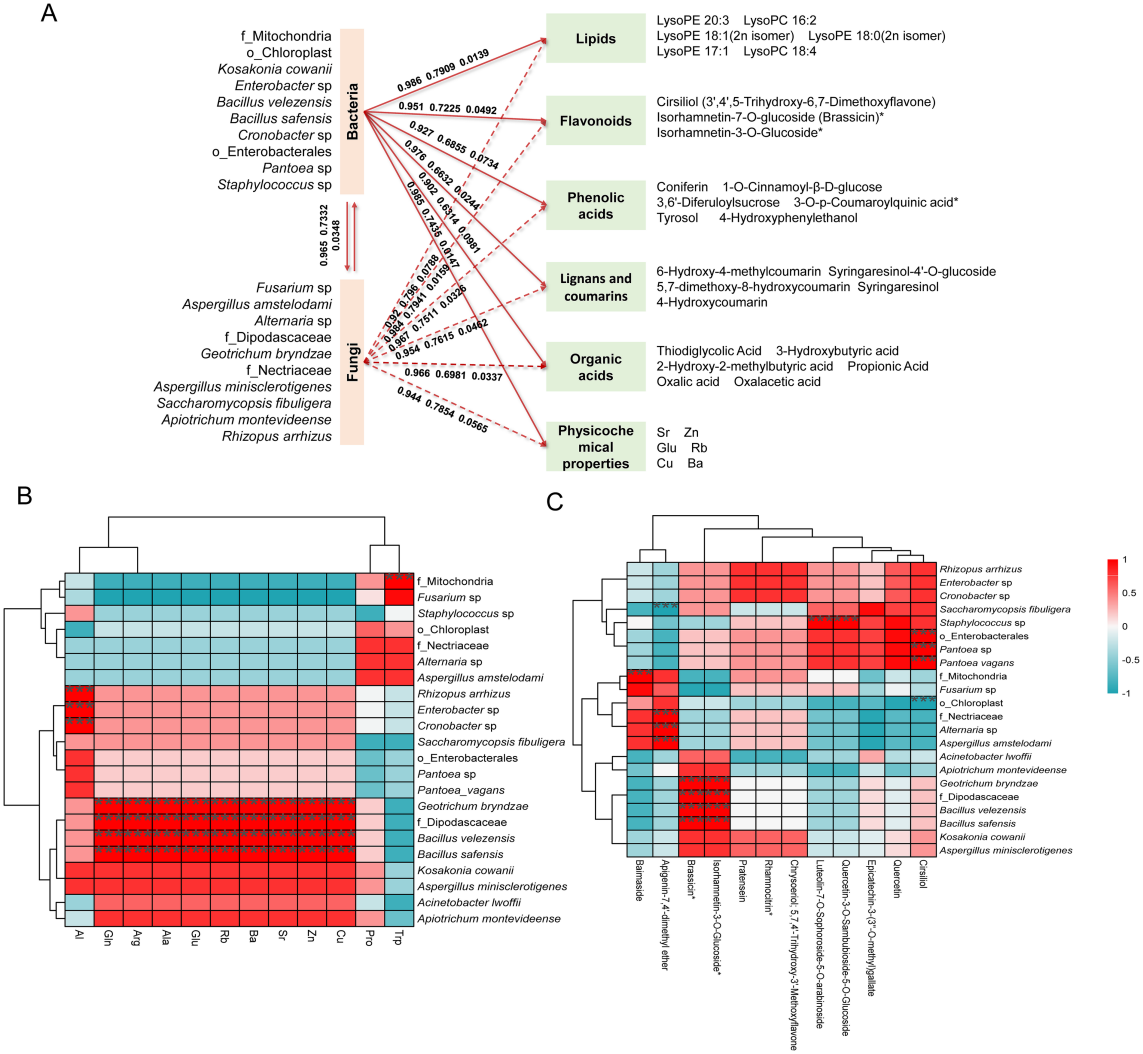
Correlation analysis and structural equation modeling of physicochemical properties, dominant species, and differential metabolites during MMF fermentation. **(A)** The structural equation model of dominant species with physicochemical properties and differential metabolites. **(B)** Correlation analysis between dominant species and physicochemical properties. **(C)** Correlation analysis between dominant species and flavonoids.

Structural equation modeling (SEM), an advanced multivariate statistical method, can evaluate complex network relationships between microorganisms and key taxonomic groups, establishing a robust and unique link between theoretical and experimental data (Banerjee et al., 2018). Therefore, we selected 12 bacterial and 10 fungal species, which were both dominant and indicator species in the MMF fermentation process, for SEM analysis. This analysis aimed to explore the potential causal relationships among these microorganisms, their physicochemical properties, and differential metabolites. Based on the primary classification, differential metabolites were divided into eight categories, and hierarchical clustering was performed on each category using the average abundance of each differential metabolite for model fitting. It was found that microorganisms had a significant correlation with five types of differential metabolites, namely lipids, flavonoids, phenolic acids, lignans and coumarins, and organic acids, which were the main active ingredients for MMF to exert its medicinal effects (Figure 6A). The total effect values of the bacteria on lipids, flavonoids, phenolic acids, lignans and coumarins, and organic acids were 0.986, 0.951, 0.927, 0.976, and 0.902, respectively, with *p*-values of 0.0139, 0.0492, 0.0734, 0.0244, and 0.0981, indicating a positive correlation. The fungi had total effect values of 0.92, 0.984, 0.967, 0.954, and 0.966 for lipids; flavonoids; phenolic acids; lignans and coumarins; and organic acids, respectively, with *p*-values of 0.0788, 0.0159, 0.0326, 0.0462, and 0.0337, demonstrating significant positive correlations. Through comprehensive comparison, the differential metabolite with the highest correlation with bacteria and fungi was flavonoids, followed by lignans and coumarins. For the physicochemical properties, the total effect value of bacteria was 0.985, with a goodness-of-fit value of 0.7435 and a *p*-value of 0.0147. For fungi, these parameters were 0.944, 0.7854, and 0.0565, indicating a significant positive correlation between bacteria and physicochemical indicators, with bacteria showing a stronger correlation than fungi. There was also a significant positive correlation between bacteria and fungi, with a total effect value of 0.965, a goodness-of-fit of 0.7332, and a *p*-value of 0.0348.

The correlation between the 22 dominant species and five inorganic elements with VIP values greater than one and six free amino acids is shown in Figure 6B. f_Mitochondria showed a highly significant positive correlation with Trp level. *Rhizopus arrhizus*, *Enterobacter* sp., and *Cronobacter* sp. exhibited highly significant positive correlations with Al. *Geotrichum bryndzae*, f_Dipodascaceae, *Bacillus velezensis*, and *Bacillus safensis* demonstrated highly significant positive correlations with multiple inorganic elements (Cu, Zn, Sr, Ba, and Rb) and free amino acids (Glu, Ala, Arg, and Gln).

**Figure 7:**
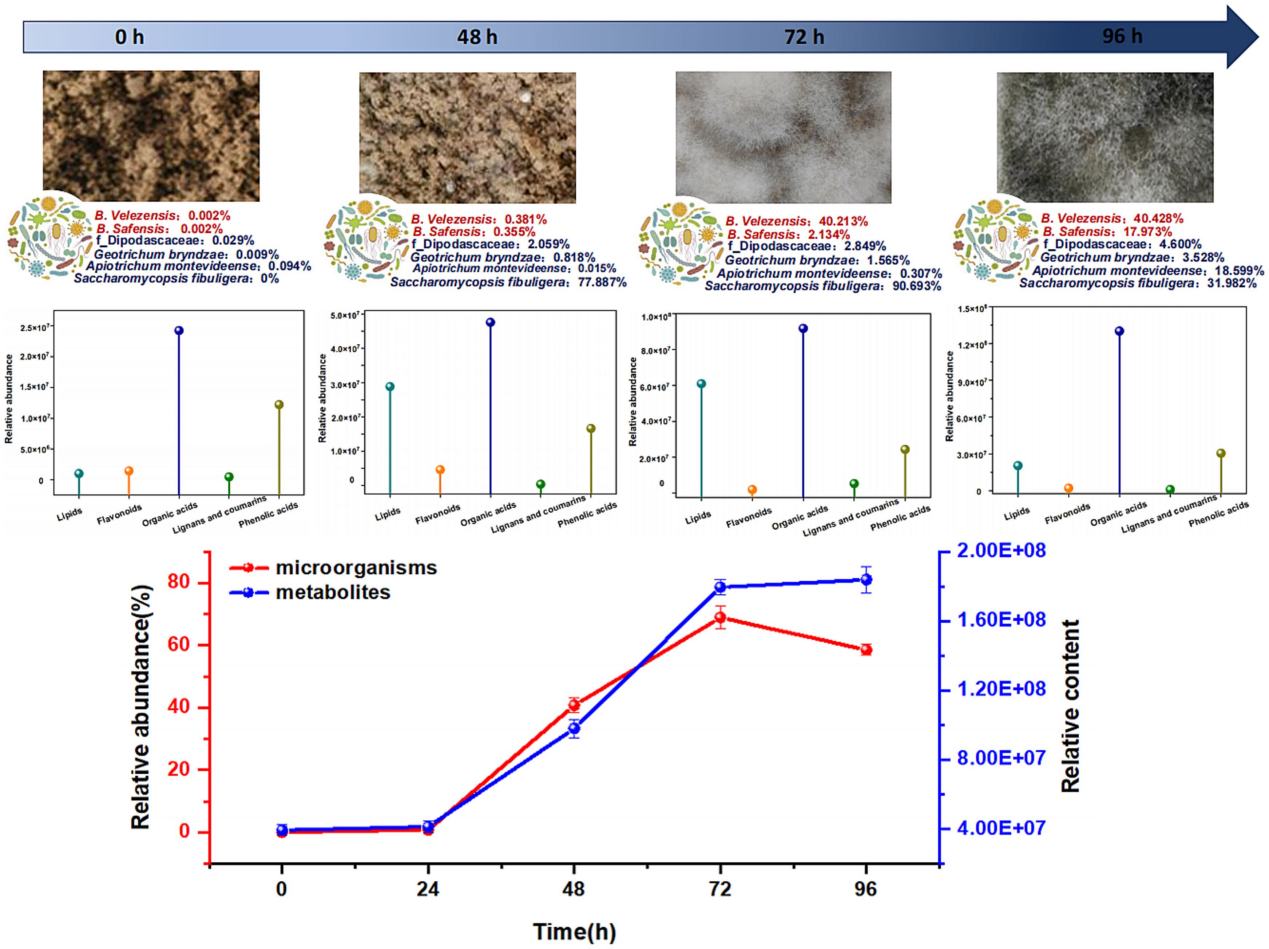
Key microbial and metabolite trends in MMF during the fermentation process.

Based on the structural equation model, Spearman’s correlation analysis was conducted to investigate the relationships between the 22 dominant species and key differential metabolites across five categories: lipids, flavonoids, phenolic acids, lignans, coumarins, and organic acids. Taking flavonoids and lignans and coumarins, which have the highest correlation with microorganisms, as examples. In flavonoids (Figure 6C), o_Enterobacterales, g_Pantoea, and *Pantoea vagans* showed highly significant positive correlations with cirsiliol, while o_Chloroplast showed a highly significant negative correlation with cirsiliol; g_Staphylococcus showed a highly significant positive correlation with quercetin-3-O-sambubioside-5-O-glucoside and luteolin-7-O-sophoroside-5-O-arabinoside; *Geotrichum bryndzae*, f_Dipodascaceae, *Bacillus velezensis*, and *Bacillus safensis* showed highly significant positive correlations with brassicin and isorhamnetin-3-O-glucoside; *Aspergillus amstelodami*, *Alternaria* sp., and f_Nectriaceae showed highly significant positive correlations with apigenin-7,4′-dimethyl ether, while *Saccharomycopsis fibuligera* showed a highly significant negative correlation with apigenin-7,4′-dimethyl ether; f_Mitochondria showed a highly significant positive correlation with Baimaside. Among lignans and coumarins (Supplementary Figure S6), *Geotrichum bryndzae*, f_Dipodascaceae, *Bacillus velezensis*, and *Bacillus safensis* showed highly significant positive correlations with syringaresinol-4′-O-glucoside and 6-hydroxy-4-methylcoumarin; *Apiotrichum montevideense* showed a highly significant positive correlation with syringaresinol; f_Mitochondria showed a highly significant positive correlation with 5,7-dimethoxy-8-hydroxycoumarin; *Acinetobacter lwoffii* showed a highly significant negative correlation with 4-hydroxycoumarin. In addition, a comprehensive comparative analysis revealed correlations between the 22 dominant species and five categories of key differential metabolites, namely lipids, flavonoids, phenolic acids, lignans and coumarins, and organic acids. The analysis showed that two bacterial species (*Bacillus velezensis*, *Bacillus safensis*) and four fungal species (*Apiotrichum montevideense*, *Geotrichum bryndzae*, f_Dipodascaceae, *Saccharomycopsis fibuligera*) were significantly correlated with these types of metabolites (Supplementary Figures S7–S9). Further analysis of the six key microorganisms and the five main differential metabolites revealed that the total abundance of the microorganisms showed an initial increase followed by a slight decrease as the fermentation time was extended, with the maximum abundance observed at 72 h. The differential metabolites exhibited similar changes (Figure 7).

### 3.8 Detailed analysis of the lipid structure during MMF fermentation

**Figure 8:**
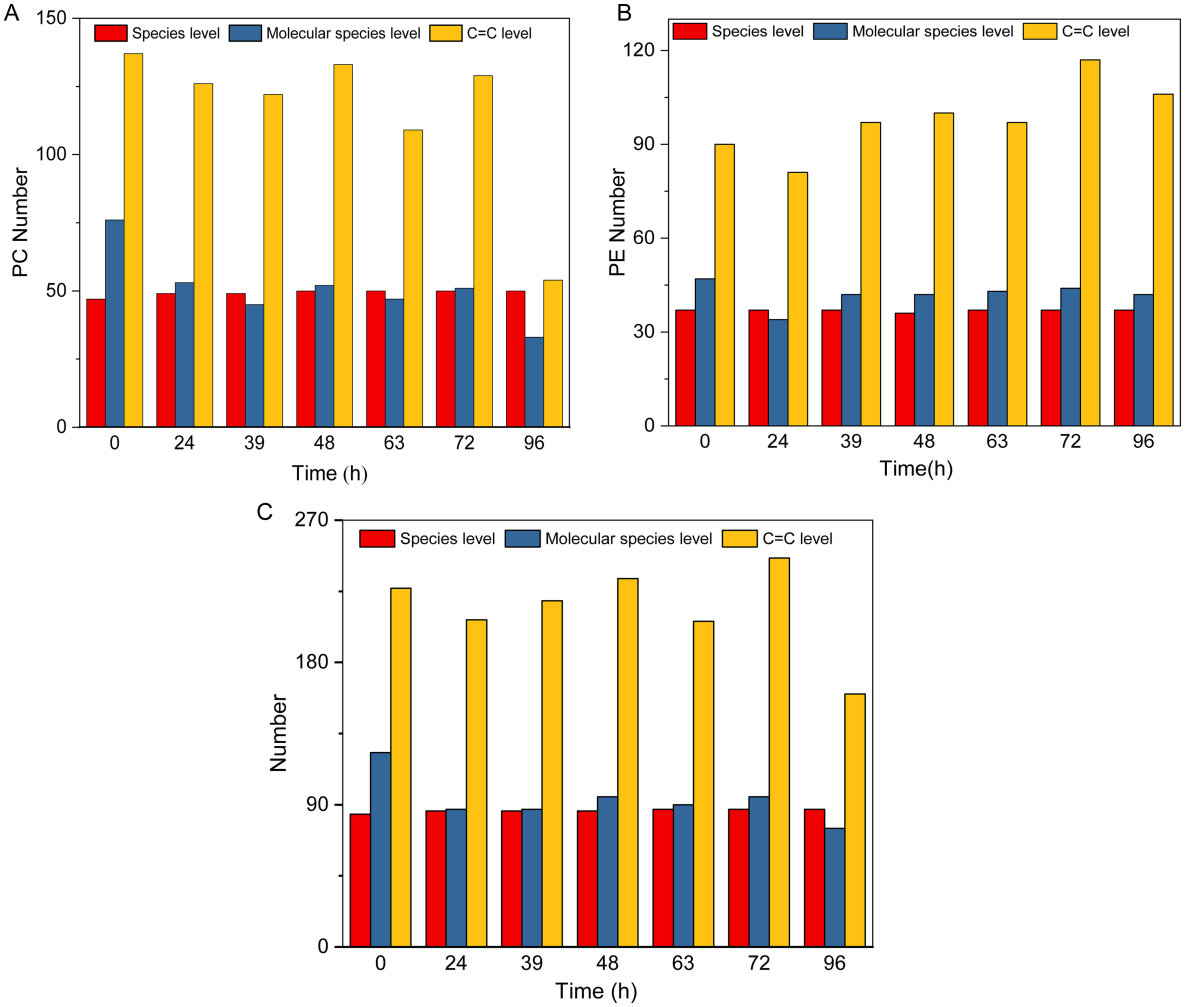
Detailed structure of lipids during MMF fermentation. **(A,B)** Types, molecular species, and levels of C=C bonds for PC and PE. **(C)** Types, molecular species, and levels of C=C bonds for total lipids.

The type and structure of lipids directly affect the fluidity, stability, and intracellular signaling of microbial cell membranes. In the analysis of upregulated differential metabolites, lipids represented the highest proportion and are crucial for the efficiency of MMF fermentation and product quality. By analyzing the types of lipids, molecular species, and positions of C=C bonds, we can not only identify specific lipid molecules and unsaturated fatty acids that play key roles during MMF fermentation and reveal their potential biological activities, such as antioxidant and anti-inflammatory effects. Therefore, in this study, we performed a detailed structural analysis of lipids during MMF fermentation, focusing on their type, molecular species, and C=C bond positions. Phosphatidylcholine (PC) and phosphatidylethanolamine (PE) are key lipid molecules in the MMF fermentation process and are known for their antioxidant properties, roles in lipid metabolism regulation, and cellular signal transduction. They significantly affect the fermentation efficiency and product quality and are enhanced or transformed by microbial metabolic activity during MMF fermentation. Structural analyses of PC and PE during MMF fermentation were conducted at the lipid type, molecular species, and C=C bond position (Figures 8A,B). Throughout the MMF fermentation process, the PC and PE types remained relatively stable. On average, PC levels were higher at 49 compared to PE levels, which averaged 37. In general, during the MMF fermentation process, the lipid types remained relatively stable, whereas the molecular species and C=C bond positions changed significantly with increasing fermentation time (Figure 8C). The number of molecular species was highest at the beginning of fermentation, reaching up to 123 species, and decreased over time, dropping to a minimum of 75 species after 96 h. The number of C=C bonds did not follow a clear pattern; it peaked at 246 species after 72 h of fermentation but fell to a minimum of 160 species after over-fermentation for 96 h. This indicates that 72 h is a critical turning point in the fermentation process, at which the accumulation of microbial metabolites reaches a relative peak, and the reducing power of MMF is at its highest, which may be an important reason for the efficacy of MMF.

## 4 Discussion

MMF is a fermented Chinese herbal medicine that has been widely used in China since the early 3rd century. MMF is also an active ingredient in several ready-to-use traditional Chinese medicines, such as Hawthorn Pills and Jiao Sanxian. During fermentation, microorganisms play a crucial role in the biotransformation, esterification, glycosylation, acetylation, and other modifications of complex chemical components in feedstocks, leading to the production of various secondary metabolites. This process involves the synergistic action of microbial communities, including *Rhizopus oryzae*, Fibroyeast, and *Aspergillus oryzae*, which significantly enhance the extraction, absorption, and utilization of active ingredients, thereby improving the efficacy of MMF. However, spontaneous fermentation presents several limitations, such as a lack of standardized quantitative parameters, reliance on empirical methods for assessing the fermentation process and quality, and the random introduction of toxin-producing species, resulting in inconsistent quality and efficacy of MMF. Therefore, it is essential to understand the dynamic changes in the composition of high-quality MMF during its fermentation maturation to enhance its quality. With the continuous advancement of modern science and technology, researchers utilize analytical tools such as thin-layer chromatography, high-performance liquid chromatography, and multi-omics techniques to investigate the chemical composition, enzyme activity, and microbial community structure during MMF fermentation. However, relying solely on a single standard is insufficient for ensuring quality control. Consequently, we conducted a joint analysis of the physicochemical properties, microorganisms, and metabolites during MMF fermentation to identify potential key factors that can enhance fermentation quality and provide greater opportunities for quality control.

Studying the dynamics of microbial communities is crucial for stabilizing the quality of MMF products. Research on dynamic changes in digestive enzyme activity during MMF fermentation has determined that the optimal fermentation time is 96 h (Zhang et al., 2022; Zhang et al., 2018). Therefore, a 96-h fermentation cycle was used in the systematic study of microbial community dynamics. The α-diversity index (Shannon) showed that bacterial diversity initially decreased, then increased, and decreased again owing to over-fermentation, whereas fungal diversity increased from 0 to 24 h, decreased from 24 to 72 h, and increased again with over-fermentation (Figures 3A,B). Changes in the diversity of the microbial community are closely related to the fermentation environment. As the fermentation time increases, variations in pH, oxygen concentration, temperature, and other environmental factors occur, leading to the death of less tolerant microorganisms and a decrease in their numbers. In addition, the growth of dominant species, accumulation of secondary metabolites, and reduction in nutrients contributed to a decrease in α-diversity. For instance, in bacteria, the proportion of *Pantoea* sp. reached its peak at 39 h of fermentation, with an abundance of 69.53%, and α-diversity dropped to its lowest. *Rhizopus arrhizus* reached its highest proportion at 39 h with an abundance of 76.43%, but it was later replaced by *Saccharomycopsis fibuligera*, which peaked at 72 h with an abundance of 90.69%. Fungal α-diversity shows a significant decrease from 24 to 72 h (Figure 3B). As fermentation continues, α-diversity begins to rise again, possibly due to the degradation of large molecules into smaller nutritional molecules, nutrient regeneration, and the breakdown or transformation of early-stage secondary metabolites, reducing toxicity and allowing some microorganisms to recover. The decline in bacterial diversity was faster than that of fungi, likely because of shorter growth cycles (Wang et al., 2022). During fermentation, species such as o_Chloroplast showed poor tolerance to the complex fermentation environment, leading to a sharp decrease in their abundance. In contrast, species such as *Pantoea* sp. and *Rhizopus arrhizus* increased in abundance but then declined because of nutrient depletion and the accumulation of metabolic products, which inhibited further growth and reproduction. *Bacillus velezensis*, *Bacillus safensis*, and *Saccharomycopsis fibuligera* increased in abundance and became predominant in the later stages of fermentation, consistent with previous research results (Wang et al., 2022).

In the analysis of physicochemical properties, the pH value of MMF fermentation showed a decreasing trend, which is consistent with previous research (Liu et al., 2023a,b). Various organic acids, such as acetic acid, lactic acid, propionic acid, and 3-hydroxybutyric acid, were detected among these metabolites. Specific metabolic byproducts are produced by fermentation microorganisms such as *Bacillus* and *Saccharomycopsis fibuligera*. The proportions of *Bacillus* and *Saccharomycopsis fibuligera* exhibited a notable upward trend, which was found to be strongly inversely related to a decrease in pH based on correlation analysis. The moisture content initially increased, then decreased, and finally increased during over-fermentation. The increase in moisture content may be primarily due to the conversion of carbon sources into water, carbon dioxide, and energy by microorganisms during fermentation. The subsequent decrease in moisture content can be attributed to the evaporation of water that exceeds its production in the later stages of fermentation (Zhang et al., 2022). Research indicates that during the growth of *Rhizopus arrhizus*, the color of the hyphae gradually changes from pure white to grayish white as they expand and mature. *Rhizopus arrhizus* is the dominant microorganism in MMF fermentation, providing a reasonable explanation for the observed changes in the appearance of MMF (Zhang et al., 2022). Inorganic elements play a crucial role in human physiological functions, with deficiencies and excesses being closely linked to health. As an important component of traditional Chinese medicine, the detection and management of inorganic elements are central to quality control and safety evaluation. According to “The People’s Republic of China Pharmacopoeia 2020 (Part IV),” the limits for heavy metals and harmful elements in MMF are specified as follows: Pb ≤5 mg/kg, Cd ≤1 mg/kg, As ≤2 mg/kg, and Cu ≤20 mg/kg. In this study, the Pb, Cd, As, and Cu contents of all samples met these standards. Multivariate statistical analysis of inorganic elements in MMF samples at different fermentation stages revealed that the main differences were Zn, Al, Cu, Sr, Ba, and Rb. These elements were positively correlated with *Rhizopus arrhizus*, *Enterobacter* sp., *Cronobacter* sp., *Geotrichum bryndzae*, f_Dipodascaceae, *Bacillus velezensis*, and *Bacillus safensis*. These results indicate that these indicator microorganisms are closely related to changes in the inorganic element content.

Changes in metabolites during MMF fermentation were identified using ultra-high-performance liquid chromatography-tandem mass spectrometry. By analyzing metabolite compositions at four key time points (0, 48, 72, and 96 h of fermentation), It was observed that the proportions of upregulated differential metabolites were highest to lowest, in the following order: lipids, amino acids and derivatives, phenolic acids, organic acids, nucleotides and derivatives, alkaloids, flavonoids, lignans, and coumarins. Through SEM analysis of these eight types of differential metabolites and 22 microorganisms that are both dominant species and indicator species, it can be known that five types of differential metabolites, namely lipids, flavonoids, phenolic acids, lignans and coumarins, and organic acids, have a significant correlation with microorganisms, and these differential metabolites all have important pharmacological activities and play a crucial role in maintaining the health of an organism. LysoPC and LysoPE in lipids can play crucial role in regulating inflammatory diseases, such as inflammatory bowel disease, by binding to G-protein-coupled receptors (Koutroumpakis et al., 2015; Tao et al., 2018). For example, polyunsaturated LysoPE can modulate the macrophage phenotype to alleviate inflammation (Makide et al., 2009), whereas LysoPC, together with sphingolipids, constitutes over 50% of the membrane phospholipids and participates in the regulation of inflammation, adipose tissue function, and obesity-related conditions (Yang and Chen, 2022). LysoPE 16:1 has antioxidant, anti-inflammatory, and cell-signaling regulatory effects (Jiang et al., 2022). Recent studies have shown that in addition to their antitussive effects, organic acids play key roles in maintaining intestinal barrier integrity, promoting energy metabolism, regulating immune responses, and balancing metabolic states. Such as 3-hydroxybutyric acid, a water-soluble endogenous small-molecule ketone body, has antioxidant and anti-inflammatory properties and can improve the energy supply of nerve cells. It can inhibit aging, oxidative stress, and inflammatory responses induced by D-galactose (Wang et al., 2024). Erucic acid, a monounsaturated *ω*-9 fatty acid, helps regulate the accumulation of long-chain fatty acids in the brain; it enhances memory function by increasing the phosphorylation of phosphatidylinositol 3-kinase, protein kinase Cζ, extracellular signal-regulated kinase, cAMP response element-binding protein, and protein kinase B in the hippocampus (Kim et al., 2016). Short-chain fatty acids not only benefit the gut but also positively affect heart health and nervous system function (Zou et al., 2023). Flavonoids, polyphenols, and coumarins are important active substances in MMF with various pharmacological effects, such as accelerated gastrointestinal motility and anti-inflammatory, anticancer, antioxidant, and immune-regulatory activities. For instance, quercetin significantly alleviates intestinal epithelial cell damage by restoring the expression of tight junction proteins ZO-1 and occludin (Carrasco-Pozo et al., 2013). The total polyphenols in MMF exhibit significant antioxidant activity (Li et al., 2023). The phenolic compound ferulic acid has been shown to markedly accelerate gastrointestinal transit and gastric emptying in a dose-dependent manner in rats (Zhang et al., 2022; Badary et al., 2006). Preliminary screening of colon cancer HT-29 cells showed that the coumarin analogue scopoletin and its structural modifiers, possess strong *in vitro* antitumour activity (Liu et al., 2012). On this basis, the Spearman method was used to explore the microorganisms significantly related to the five main differential metabolites, including bacteria (*Bacillus velezensis*, *Bacillus safensis*) and fungi (*Apiotrichum montevideense*, *Geotrichum bryndzae*, f_Dipodascaceae, *Saccharomycopsis fibuligera*). Further research has found that the content of five main differential metabolites and the abundance of six main microorganisms change in a similar trend with the fermentation time, reaching their peaks at 72 h. This indicates that 72 h is a crucial stage for enriching pharmacodynamic components, which is of great significance for the quality and pharmacodynamic effects of MMF. These results delineate the patterns of microbial community succession during MMF fermentation and their correlations with physicochemical properties and differential metabolites. This information will serve as a foundational dataset for elucidating the fermentation process and establishing quality standards for MMF.

## 5 Conclusion

This study systematically investigated the dynamic changes in physicochemical properties, microbial communities, and broad-target metabolite profiles of MMF during fermentation under strictly controlled temperature and humidity conditions (32°C, 77.0%RH), based on traditional fermentation processes. Through physicochemical analysis, six characteristic elements (Zn, Al, Cu, Sr, Ba, Rb) and seven characteristic free amino acids (Glu, Ala, Arg, Pro, Trp, Gln, Lys) were identified as key indicators of the fermentation process. Microbial community analysis revealed a distinct succession pattern: the dominant bacterial species shifted from o_Chloroplast to *Pantoea* sp. and subsequently to *Bacillus velezensis*, while the dominant fungal species transitioned from *Alternaria* sp. to *Rhizopus arrhizus* and finally to *Saccharomycopsis fibuligera*. Structural equation modeling and Spearman’s correlation analysis demonstrated significant associations between microbial succession, physicochemical changes, and the accumulation of active components. These findings provide valuable insights for distinguishing fermentation stages, enhancing microbial activity, monitoring fermentation processes, and achieving precise quality control in MMF production.

## Data Availability

The original contributions presented in the study are included in the article/Supplementary material, further inquiries can be directed to the corresponding author.
