# Supplementary material for: Comprehensive analysis on the regulatory mechanism of active ingredient accumulation during fermentation process of Massa Medicata Fermentata: microbe and metabolic profiles
Source: Front Microbiol. 2025 Mar 12;16:1548427. doi: 10.3389/fmicb.2025.1548427 (PMC11936943; doi:10.3389/fmicb.2025.1548427)
Supplement: Supplementary file 1 [file Data_Sheet_1.pdf]

Supplementary Material

List of Captions

Supplementary Figure 1. Optimization of the Traditional Fermentation Process of MMF.

Supplementary Figure 2. PCA scatter plot of 21 inorganic elements.

Supplementary Figure 3. Dominant genera of bacteria at genus level.

Supplementary Figure 4. Dominant genera of fungi at genus level.

Supplementary Figure 5. MRM plots of mixed control solution and MMF test solution.

Supplementary Figure 6. Correlation analysis between dominant strains and lignans and coumarins.

Supplementary Figure 7. Correlation analysis between dominant strains and Lipids.

Supplementary Figure 8. Correlation analysis between dominant strains and organic acids.

Supplementary Figure 9. Correlation analysis between dominant strains and phenolic acids.

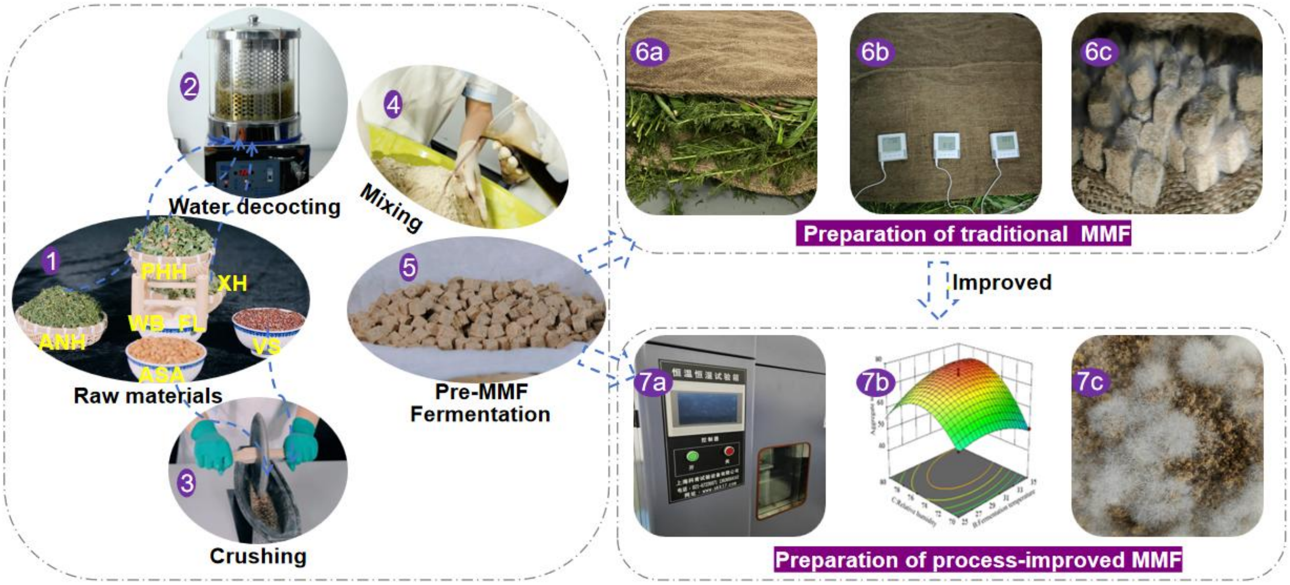

ANH, Artemisiae annuae herba; PHH, Polygoni hydropiperis Herba; XH, Xanthii herba; WB, wheat bran; FL, Flour; ASA, Armeniacae semen amarum; VS, Vignae semen

Supplementary Figure 1. Optimization of the Traditional Fermentation Process of MMF.

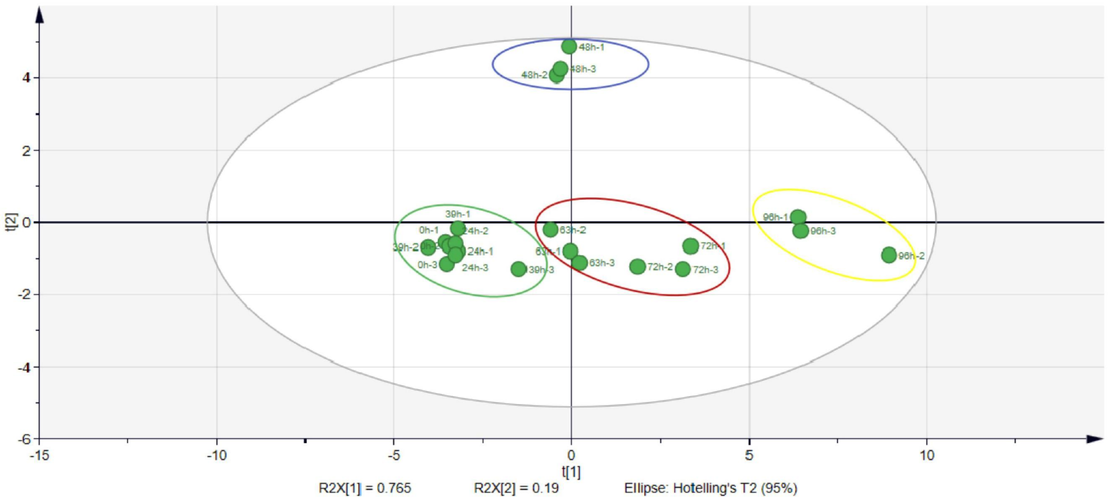

Supplementary Figure 2. PCA scatter plot of 21 inorganic elements.

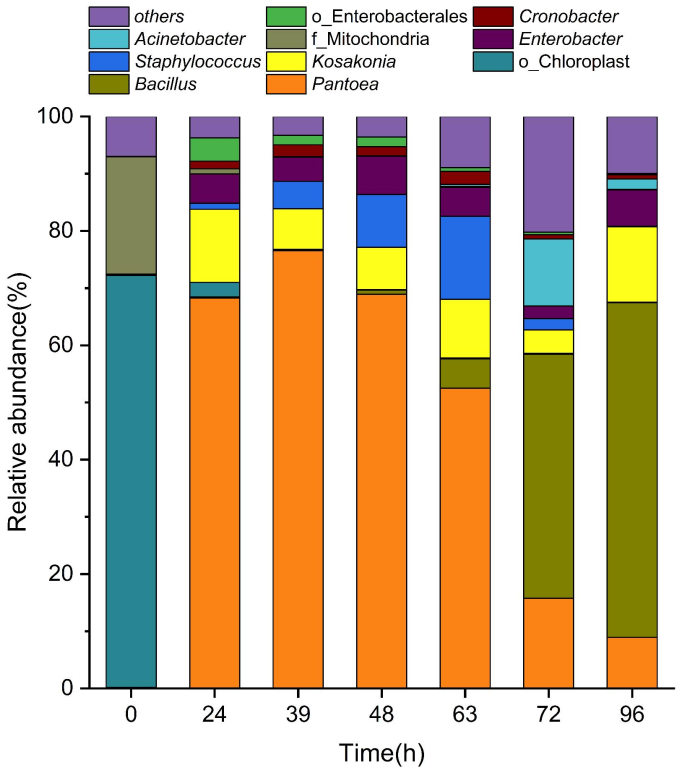

Supplementary Figure 3. Dominant genera of bacteria at genus level.

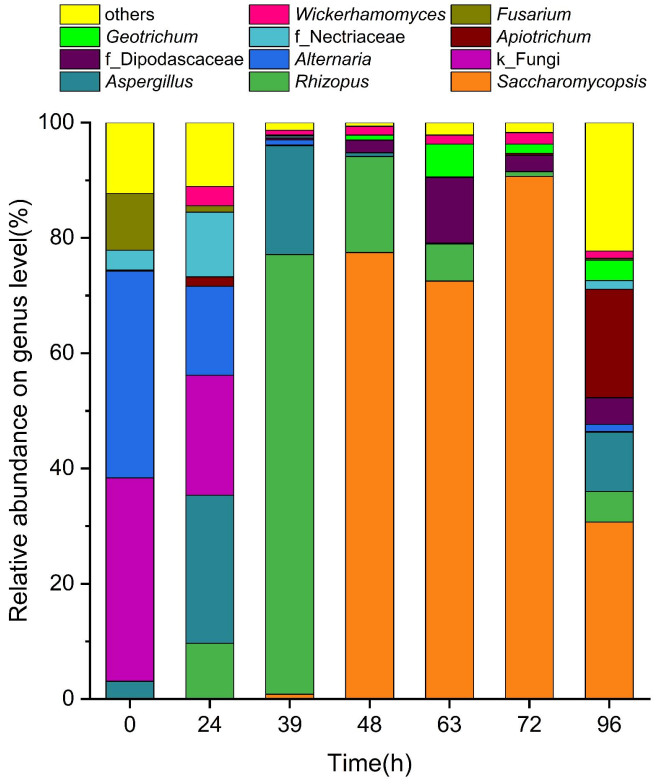

Supplementary Figure 4. Dominant genera of fungi at genus level.

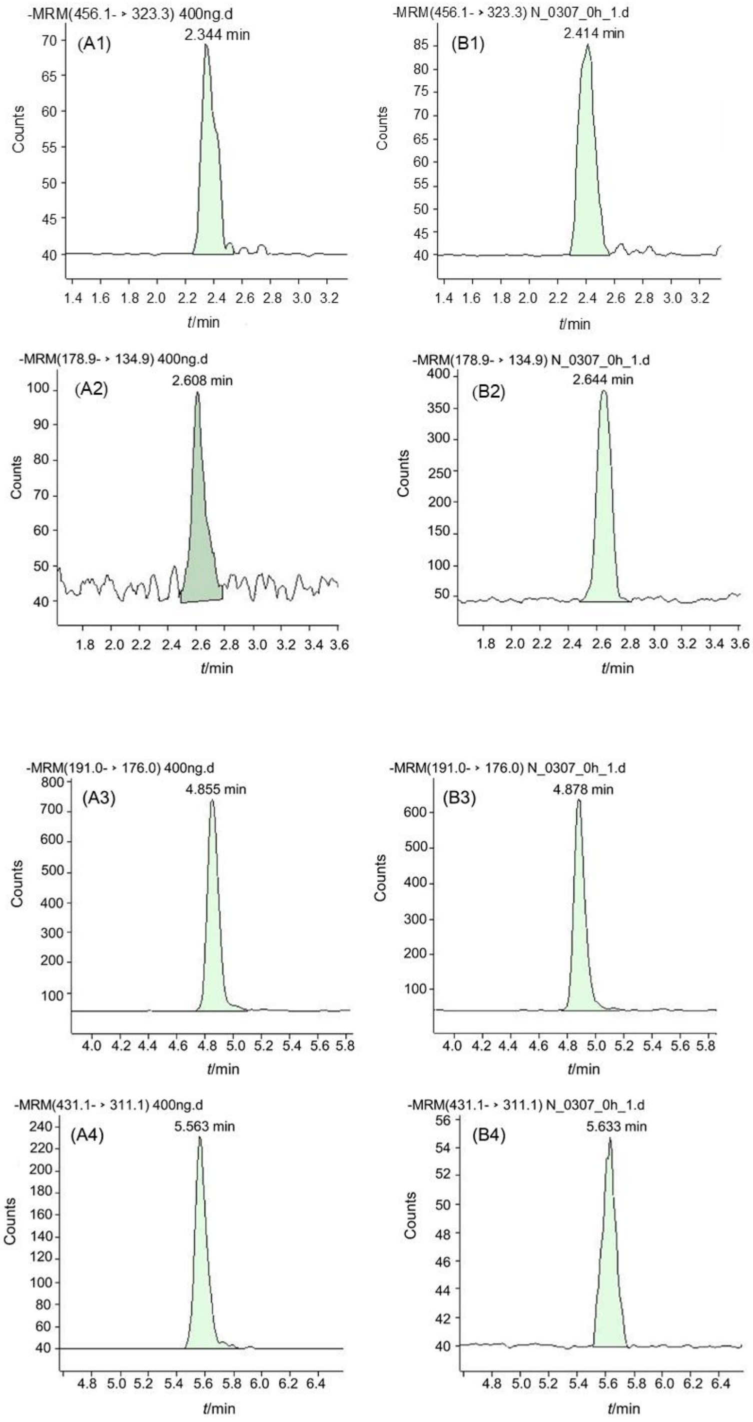
-MRM(456.1- › 323.3) 400ng.d
(A1)
2.344 min
-MRM(456.1- › 323.3) N_0307_0h_1.d
(B1)
2.414 min
-MRM(178.9- › 134.9) 400ng.d
(A2)
2.608 min
-MRM(178.9- › 134.9) N_0307_0h_1.d
(B2)
2.644 min
-MRM(191.0- › 176.0) 400ng.d
(A3)
4.855 min
-MRM(191.0- › 176.0) N_0307_0h_1.d
(B3)
4.878 min
-MRM(431.1- › 311.1) 400ng.d
(A4)
5.563 min
-MRM(431.1- › 311.1) N_0307_0h_1.d
(B4)
5.633 min
Counts
t/min

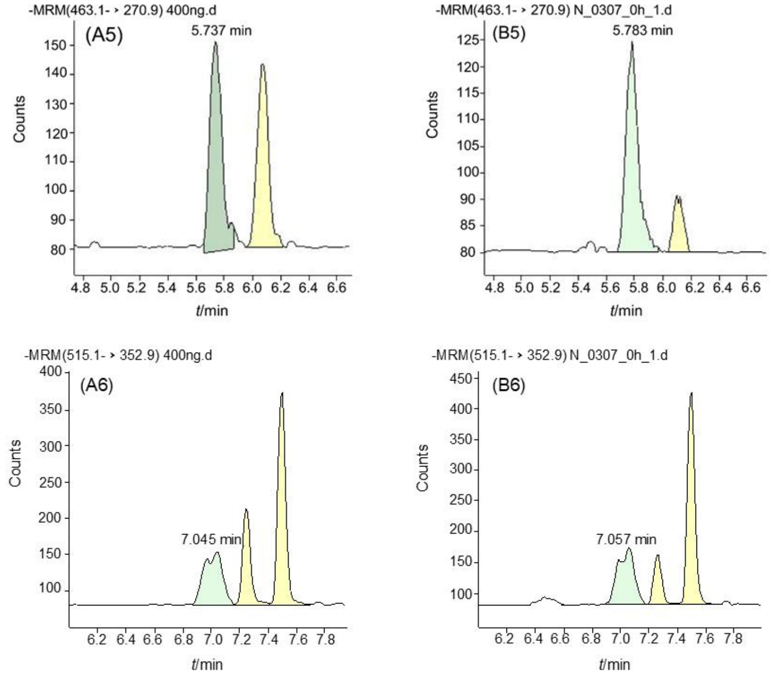
-MRM(463.1- › 270.9) 400ng.d
(A5)
5.737 min
Counts
t/min
-MRM(463.1- › 270.9) N_0307_0h_1.d
(B5)
5.783 min
Counts
t/min
-MRM(515.1- › 352.9) 400ng.d
(A6)
7.045 min
Counts
t/min
-MRM(515.1- › 352.9) N_0307_0h_1.d
(B6)
7.057 min
Counts
t/min

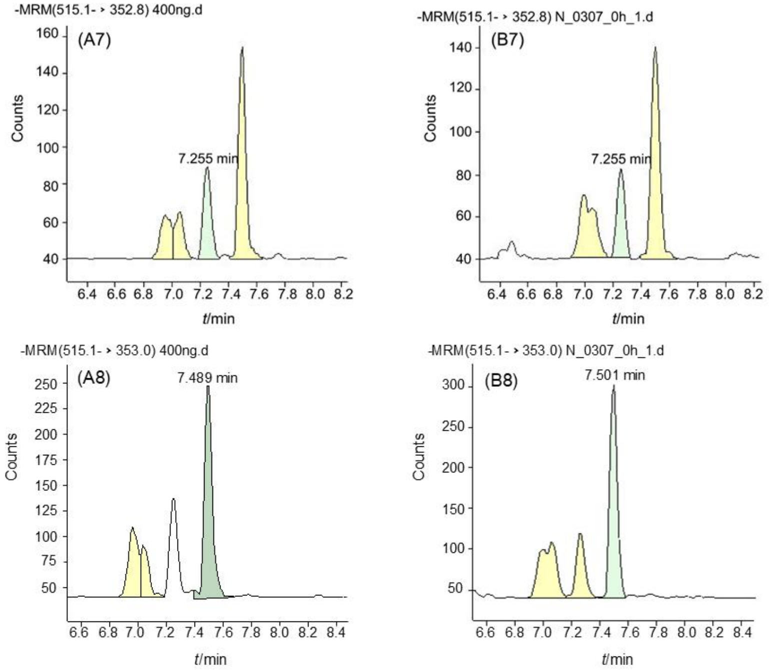
-MRM(515.1- › 352.8) 400ng.d
(A7)
7.255 min
Counts
t/min
-MRM(515.1- › 352.8) N_0307_0h_1.d
(B7)
7.255 min
Counts
t/min
-MRM(515.1- › 353.0) 400ng.d
(A8)
7.489 min
Counts
t/min
-MRM(515.1- › 353.0) N_0307_0h_1.d
(B8)
7.501 min
Counts
t/min

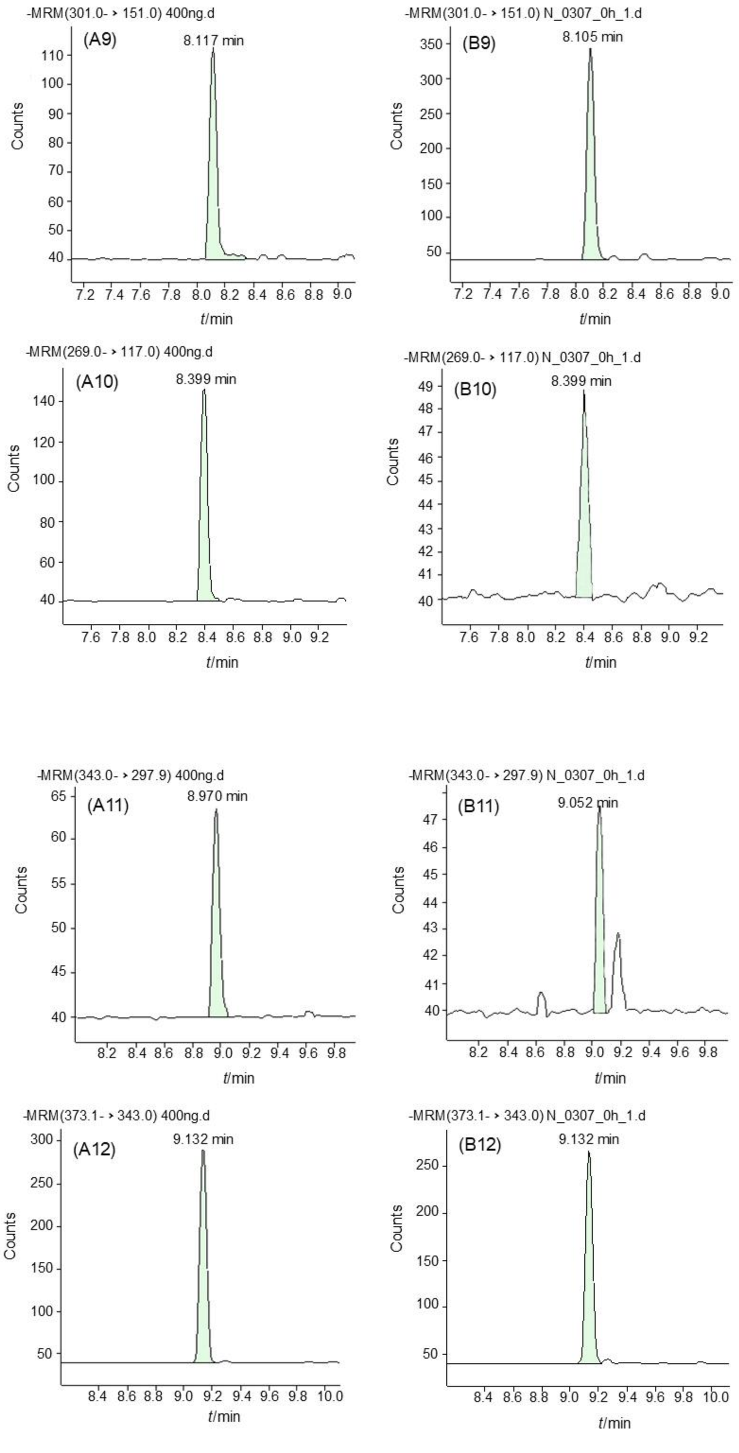

Supplementary Figure 5. MRM plots of mixed control solution and MMF test solution.

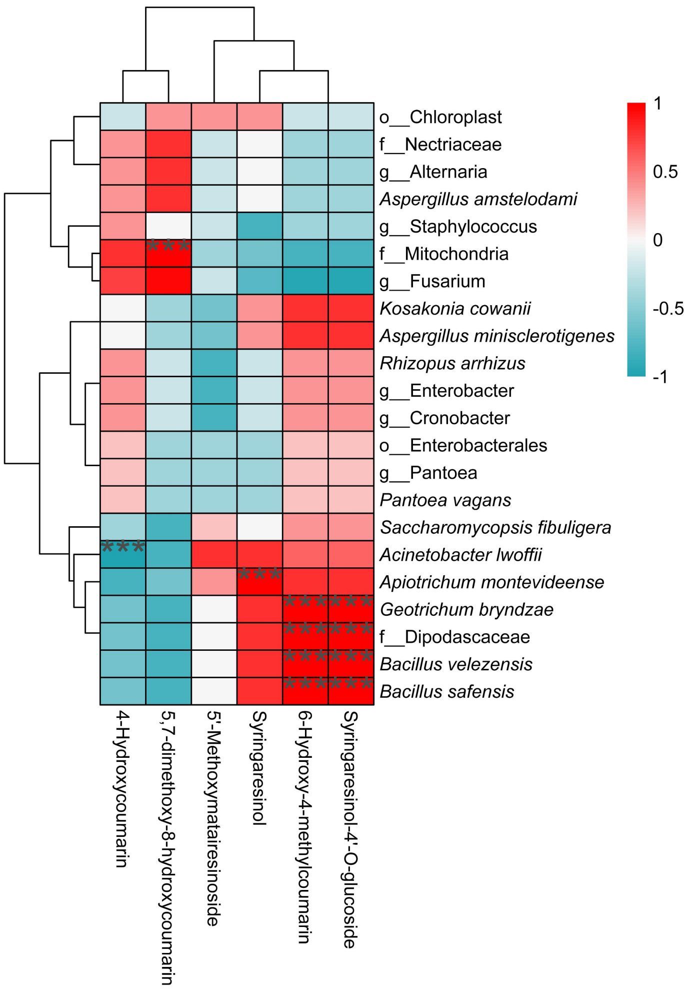

Supplementary Figure 6. Correlation analysis between dominant strains and lignans and coumarins.

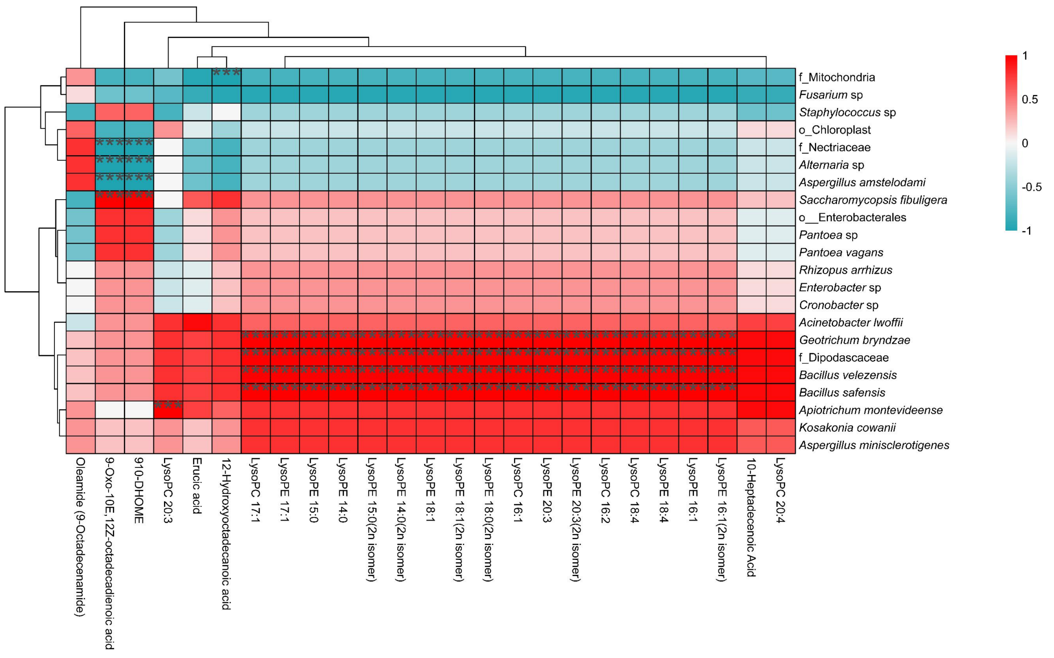

Supplementary Figure 7. Correlation analysis between dominant strains and Lipids.

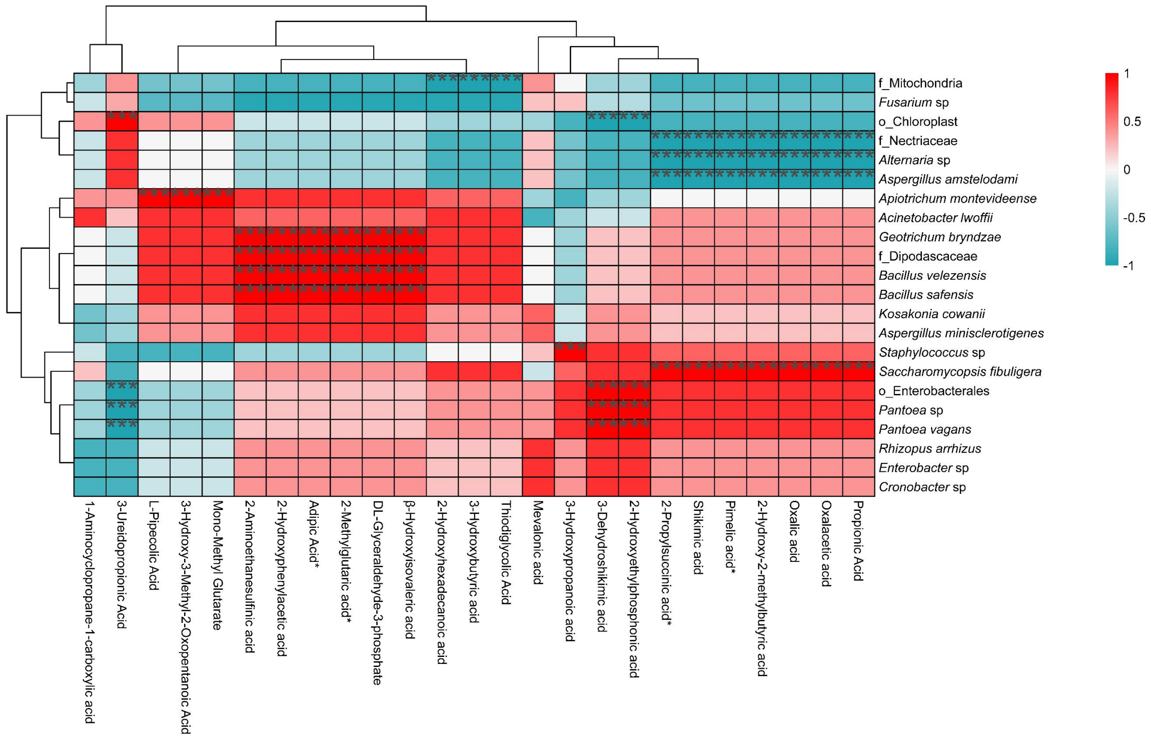

Supplementary Figure 8. Correlation analysis between dominant strains and organic acids.

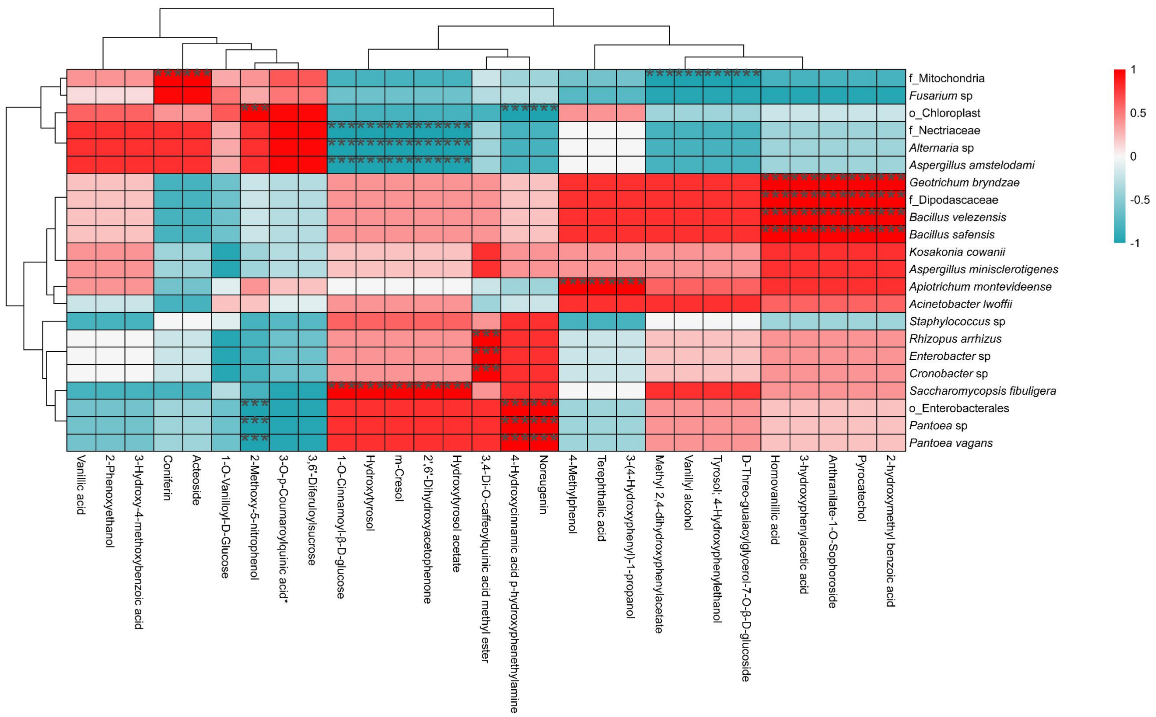

Supplementary Figure 9. Correlation analysis between dominant strains and phenolic acids.
